# Supplementary material for: Antibiotic Skeletal Diversification via Differential Enoylreductase Recruitment and Module Iteration in trans-Acyltransferase Polyketide Synthases
Source: J Am Chem Soc. 2024 Feb 23;146(9):6114–24. doi: 10.1021/jacs.3c13667 (PMC10921412; doi:10.1021/jacs.3c13667)
Supplement: Supplementary file 1 — ja3c13667_si_001.pdf [file ja3c13667_si_001.pdf]

Supporting Information

Antibiotic skeletal diversification via differential enoylreductase recruitment and module iteration in *trans*-acyltransferase polyketide synthases

Xinyun Jian[1,2,3,4,‡], Fang Pang[1,‡, ǂ], Christian Hobson[1ǂ], Matthew Jenner[1,2], Lona M. Alkhalaf[1], and Gregory L. Challis[1,2,3,4*]

[1]Department of Chemistry, University of Warwick, Coventry CV4 7AL, UK

[2]Warwick Integrative Synthetic Biology Centre, University of Warwick, Coventry CV4 7AL, UK

[3]Department of Biochemistry and Molecular Biology, Biomedicine Discovery Institute, Monash University, Clayton, VIC 3800, Australia

[4]ARC Centre of Excellence for Innovations in Protein and Peptide Science, Monash University, Clayton, VIC 3800, Australia

[‡]These authors contributed equally

*Corresponding Author; Email: G.L.Challis@warwick.ac.uk

[ǂ]Current addresses: Willow Biosciences, 319 N Bernardo Ave #4, Mountain View, CA 94043, USA (C.H.), Department of Chemistry, Johns Hopkins University, 3400 N. Charles Street, Baltimore, MD 21218, USA (F.P.)

Methods

Creation and modification of protein overproduction constructs

DNA fragments encoding the proteins of interest were amplified by PCR from *B. gladioli* BCC0238 gDNA using Q5® Hot Start High-Fidelity DNA polymerase (New England Biolabs) and amplimers were separated on a 0.8% agarose gel. The desired DNA fragments were excised from the gel, purified using the GeneJET Gel Extraction Kit (Thermo Fisher Scientific) and inserted into pET151 (Thermo Fisher Scientific), pETSUMO (Thermo Fisher Scientific), or pET28a(+) (New England Biolabs) following the manufacturer's instructions. The EtnK AT domain overproduction construct (pET24a-*etnK*) was synthesized by Epoch Life Sciences and was designed to incorporate an N-terminal octa-histidine tag. Mutant constructs pET-SUMO-*gbnE*-H198V and pET28a(+)-*gbnD2*_Module 6-S941A were generated using the Q5® Site-Directed Mutagenesis Kit (New England Biolabs). Ligation reactions were used to transform *E. coli* TOP10 cells and single colonies were picked and grown overnight at 37 °C and 180 rpm in LB medium containing 50 mg/mL kanamycin or 100 mg/mL ampicillin, as appropriate. Plasmids were isolated from overnight culture using the GeneJET Plasmid Miniprep Kit (Thermo Fisher Scientific) and verified by sequencing. The primers used to amplify an mutagenize protein-encoding DNA fragments are listed in Table S1.

Table S1: Primers used for amplification and mutagenesis of DNA fragments encoding recombinant proteins used in the study.

Construct	Primer
pET151-*gbnD1*_ER-ACP	For: *CACC*CCGGAACCGGCTGAGGCG
	Rev: *TCA*CGGCGCATGCGTCTCGCT
pET151-*gbnD1*_ER	For: *CACC*GCTGCGCCTTCGCCTTCC
	Rev: *TCA*CGGCGCATGCGTCTCGCT
pET151-*gbnD2*_ACP5	For: *CACC*CCGAATCCAGCGTCGCTTTCG
	Rev: *TCA*GACCTCCGCCTTGGCCGTCTC
pET28a(+)-*gbnD1*_ACP3	For: *ATAT*CATATGACCGCCACGGTTCCGACC
	Rev: *ATAT*GAATTCTCAGATCGCGGGCCCGGAGG
pET28a(+)-*gbnD2*_ACP4	For: *ATAT*CATATGCCGGCAACGCAACGCGCC
	Rev: *ATAT*GAATTCTCACGCGCTCGCTGTCGCAGG
pET28a(+)-*gbnD4*_ACP10	For: *ATAT*CATATGGCCCAGGCGAAGCCGGTT
	Rev: *ATAT*AAGCTTTCAGCCCTCGGGCAGGCCAA
pET28a(+)-*gbnD2*_Module 5	For: *ATAT*GCTAGCGCGGGCGCGAGGACGATG
	Rev: *ATAT*AAGCTTTCAGACCTCCGCCTTGGCCGT
pET28a(+)-*gbnD2*_Module 5-C209A	For: GGACACCATGgcgTCGTCGTCGCTGACGG
	Rev: ATCGCGATGCTGGGACCG
pET28a(+)-*gbnD2*_Module 6	For: *ATA*GCTAGCGACGGCGAGACGGCCAAG

	Rev: *ATA*AAGCTTTCACGGGGCGTTTCCGGTCTT
pET28a(+)-*gbnD2*_Module 6-S941A	For: ACTGGGCATCGAT*g*CGGTGGTCG
	Rev: TCGGCGAACGGCGTGTCG
pET-SUMO-*gbnE*-H198V	For: TTCGGGCGGT*gtT*ACCGATCGC
	Rev: TCGGCCTCCACGCAGATGTC
pET-SUMO-*gbnE*	For: ATGGCAATGATTACCGCA
	Rev: CTATTGCCTCTGGAGTTGAAGTGA
pET28a-MBP-TEV-*etnL*	For: CGCGGATCCATGATTACCGCCAAAAGTCT
(codon optimized)	Rev: CCGCTCGAGTTAACTGCTAACGCTCTGTG
pET28a-*etnE*_ACP5	For: ATATCATATGAGTCCCGGTGATCTGGCG
(codon optimized)	Rev: ATATAAGCTTTCAGGCGCTCACGGCGGTGGC
pET28a-*etnG*_ACP10	For: ATATCATATGACGCCGGCGAGCGAGCAG
	Rev: ATATGAATTCTCAGGCCGCGACCTCCGCTCG

Protein overproduction and purification

Single colonies of *E. coli* BL21 (DE3) transformants containing the expression constructs were picked and grown in 10 mL LB containing 50 mg/mL kanamycin or 100 mg/mL ampicillin, as appropriate, at 37 °C and 180 rpm overnight. 1L LB containing 50 mg/mL kanamycin or 100 mg/mL ampicillin, as appropriate, was separately inoculated with each 10 mL overnight culture and grown at 37 °C and 180 rpm until an optical density at 600 nm of 0.6 was reached. 0.5 mM IPTG was added to each culture and growth was continued at 15 °C and 180 rpm for 18-20 h. The cells from each culture were harvested by centrifugation (4,000 *g* at 4 °C for 15 min), separately resuspended in 10 mL of loading buffer (20 mM Tris, 300 mM NaCl, 20 mM imidazole, pH 8.0), and lysed using a cell disruptor (Constant Systems). Each lysate was centrifuged (17,000 *g* at 4 °C for 45 min) and the supernatant was filtered and separately loaded onto a HiTrap Chelating Column (GE HealthCare) that had been pre-loaded with 0.1 M $NiSO_4$ followed by equilibration with loading buffer (20 mM Tris, 300 mM NaCl, 20 mM imidazole, pH 8.0). The loaded columns were washed with 15 mL loading buffer (20 mM Tris, 300 mM NaCl, 20 mM imidazole, pH 8.0) and the proteins were eluted in a stepwise manner using loading buffer containing increasing concentrations of imidazole: 50 mM (5 ml), 100 mM (3 ml), 200 mM (3 ml) and 300 mM (3ml). The presence of the proteins of interest was confirmed by SDS-PAGE and each was further purified by gel filtration using a Superdex 200pg column (GE Healthcare), as required. Fractions containing the same purified protein were combined, exchanged into storage buffer (20 mM Tris, 300 mM NaCl, 10% glycerol, pH 7.4), concentrated using a VivaSpin centrifuge filter (GE Healthcare) with an appropriate molecular weight cut off (MWCO), snap-frozen in liquid nitrogen and stored at -80 °C.

***In vitro* assays:**

Enoyl reduction assay for ACP domain bound substrate

The *apo*-ACP domains excised from modules 3, 5, 10 and 13 or contained within the module 1 ACP-ER didomain construct were loaded with crotonyl-Ppant by incubating 100 μM of each protein with 2μM Sfp (purified recombinant PPtase from *Bacillus subtilus*),[1] 10mM $MgCl_2$ and 800 μM crotonyl-CoA in storage buffer (20 mM Tris, 300 mM NaCl, 10% glycerol, pH 7.4) in a total volume of 50 μl. After 30 min incubation at room temperature, UHPLC-ESI-Q-TOF-MS was used to confirm each loading reaction was complete.

For the module 1 ACP-ER didomain enoyl reduction assay, 50 μM crotonylated-*holo*-ACP-ER didomain was incubated with 500 μM NADPH in storage buffer in a total volume of 50 μl. The control reaction was conducted under the same conditions except NADPH was omitted.

For the enoyl reduction assays employing the excised ACP domains, 50 μM of the requisite crotonylated-*holo*-ACP domain was incubated with 50 μM of the module 1 ER domain, GbnE or GbnE-H198V and 500 μM NADPH in storage buffer (50 μl total volume). Control reactions were conducted under the same conditions lacking the module 1 ER domain, GbnE or GbnE-H198V. The reactions were incubated at room temperature for 5h and the nature of the acyl group attached to each ACP domain was analyzed using UHPLC-ESI-Q-TOF-MS/MS (Ppant ejection).

Module 5 iteration assays

KS domain acylation

The 2,4-hexadienoyl unit was attached to the KS domain by incubating 50 μM module 5 with 1 mM 2,4-hexadienoyl NAC thioester **7** in storage buffer (20 mM Tris, 300 mM NaCl, 10% glycerol, pH 7.4) in a total volume of 50 μl. After 3 h incubation at room temperature, the protein was concentrated to 150 μM using a VivaSpin concentrator with a MWCO of 50 kDa (GE Healthcare). In the control reaction, the mutant construct module 5-C209A was used instead of the wild type protein.

Iteration assay with intact module 5

50 μM hexadienoylated-module 5 was incubated with 1 μM Sfp,[1] 10 mM $MgCl_2$, 400 μM malonyl-CoA, 25 μM EtnK AT domain and 800 μM NADPH in the presence / absence of 0.5-50 μM GbnE/GbnE-H198V in storage buffer (20 mM Tris, 300 mM NaCl, 10% glycerol, pH 7.4) in a total volume of 50 μl at room temperature for 20 h. The assay was monitored via UHPLC-ESI-Q-TOF-MS analysis of the intact protein and products were cleaved from the ACP domain via addition of cysteamine to a final concentration of 0.2 M. After 20 h incubation at 4 °C, the mixture was extracted with EtOAc (2 x 1 mL) and the combined extracts were evaporated to

dryness. The residue was dissolved in 100 µL of MeOH and analyzed by UHPLC-ESI-Q-TOF-MS. The instrumentation, elution conditions and settings used for these analyses were the same as those used for analysis of the metabolites produced by *B. gladioli* wild type and in-frame deletion mutants (see below).

***Iteration assay employing module 5 ACP domain* in trans**

The excised module 5 ACP domain was loaded with malonyl-Ppant by incubating 200 µM protein with 2µM Sfp,[1] 10 mM $MgCl_2$ and 800 µM malonyl-CoA in storage buffer in final volume of 50 µl for 30 min at room temperature and UHPLC-ESI-Q-TOF-MS was used to confirm the reaction had gone to completion. 50 µM hexadienoylated-module 5 was incubated with 100 µM malonyated-module 5 *holo*-ACP domain and 800 µM NADPH in the presence / absence of 0.5-50 µM GbnE/GbnE-H198V in storage buffer (final volume 50 µl) at room temperature for 20 h. The species attached to the excised module 5 ACP domain after 2, 5 and 20 h were assessed using UHPLC-ESI-Q-TOF-MS analyses of intact proteins and cysteamine cleavage adducts (as described in the section above on the iteration assay with intact module 5).

Assay of transacylation from the module 5 ACP domain to the module 6 KS domain

The module 5 ACP domain was loaded with 2,4-hexadienoyl-Ppant or 4-hexaenoyl-Ppant by incubating 100 µM protein with 2 µM CoaA, 2 µM CoaC, 2 µM CoaD, 2 µM Sfp,[2] 10 mM $MgCl_2$, 5 mM ATP and 1 mM 2,4-hexadienoyl pantetheine thioester **17** or 4-hexenoyl pantetheine thioester **18** in storage buffer (20 mM Tris, 300 mM NaCl, 10% glycerol, pH 7.4) in a total volume of 50 µl for 1.5 h at room temperature. UHPLC-ESI-Q-TOF-MS was used to confirm the reactions had gone to completion. The excess pantetheine thioesters were removed by two rounds of 10-fold dilution with storage buffer and concentration using a VivaSpin centrifuge filter with a MWCO of 5 kDa (GE Healthcare). 100 µM of the hexadienoylated or hexenoylated-module 5 *holo*-ACP domain was incubated with 300 µM of the module 6 KS-ACP-ACP-S941A tridomain in storage buffer (total volume 50 µl) for 8h at room temperature. The acylation state of the module 5 ACP domain after 2 h, 4 h, 6 h and 8 h was assessed by UHPLC-ESI-Q-TOF-MS.

Module 6 elongation assays

The KS domain of the module 6 KS-ACP-ACP(S941A) tri-domain was acylated with a 2,4-hexadienoyl or a 4-hexenoyl unit by incubating 50 µM tri-domain with 1mM 2,4-hexadienoyl NAC thioester **7** or 4-hexenoyl NAC thioester **19**, respectively, in storage buffer (20 mM Tris, 300 mM NaCl, 10% glycerol, pH 7.4) in a total volume of 50 µl at room temperature for 3 h. Acylation of the tri-domain was confirmed by UHPLC-ESI-Q-TOF-MS analysis. The acylated tri-domains were incubated with 1 µM Sfp,[1] 500 µM malonyl-CoA and 10mM $MgCl_2$ in

storage buffer (total volume of 50 µl). Control reactions were conducted under the same conditions except that the 2,4-hexadienoyl and 4-hexenoyl NAC thioesters were omitted. After 6 h incubation at room temperature the acylation state of the tri-domains was assessed using UHPLC-ESI-Q-TOF-MS.

Intact protein analysis using UHPLC-ESI-Q-TOF-MS

Purified recombinant proteins and proteins of interest in enzymatic activity assays were analysed using a Bruker MaXis II ESI-Q-TOF-MS connected to a Dionex 3000 RS UHPLC fitted with an ACE C4-300 RP column (100 x 2.1 mm, 5 µm, 30 °C). The column was eluted with a linear gradient of 5%-100% MeCN containing 0.1% formic acid at a flow rate of 0.2 ml/min for 30 min. The mass spectrometer was operated in positive ion mode with a scan range of 200–3000 *m/z*. Source conditions were: end plate offset at −500 V; capillary at −4500 V; nebulizer gas (N_2) at 1.8 bar; dry gas (N_2) at 9.0 L min^{-1}; dry temperature at 200 °C. Ion transfer conditions were: ion funnel RF at 400 Vpp; multiple RF at 200 Vpp; quadrupole low mass at 300 *m/z*; collision energy at 8.0 eV; collision RF at 2000 Vpp; transfer time at 110.0 µs; pre-pulse storage time at 10.0 µs.

Construction and complementation of *in-frame* gene deletion mutants

In-frame gene deletions were constructed in *Burkholderia gladioli* BCC1622 (because the original gladiolin producer *B. gladioli* BCC0238 was not suitable for genetic manipulation using this system).[3] The primers listed in Table S2 were used to amplify the 5' and 3' regions flanking each gene. The resulting amplimers were cloned into pGPI-SceI and transferred into *E. coli* SY327 by electroporation. The integrity of the resulting plasmids was confirmed by sequencing and they were mobilized into *Burkholderia gladioli* BCC1622 by tri-parental mating.[4] Exconjugants were selected for using 150 µg/ml trimethoprim and 600 U/ml polymyxin B and subjected to PCR screening to identify single crossover mutants using the primers listed in Table S2. A confirmed single crossover mutant for each gene of interest was transformed with pDAI-SceI by tri-parental mating. Exconjugants were selected for using 200 µg/ml tetracycline and 600 U/ml polymyxin B and subjected to PCR screening to identify double crossover mutants using the primers listed in Table S2. The resulting mutants were confirmed as trimethoprim sensitive. Finally, the pDAI-SceI plasmid was removed from one of the double crossover mutants by growing it on M9 minimal medium containing 15% sucrose. Single colonies were picked and confirmed to be sensitive to tetracycline.

For in *trans* complementation of deletion mutants, the gene of interest was amplified using the primers listed in Table S2. The resulting amplimers were cloned into pMLBAD and used to transform *E. coli* Top10 by

electroporation. The integrity of the resulting plasmids was confirmed by sequencing and they were mobilized into the appropriate in-frame deletion mutant of *Burkholderia gladioli* BCC1622 by tri-parental mating as described above.[4] Exconjugants were selected for using 150 µg/ml trimethoprim and 600 U/ml polymyxin B and confirmed by Tp^R phenotype and PCR with the primer pairs used for initial amplification of each gene.

Table S2: Primers for gene in-frame deletion and complementation

**Construct**	**Primer (restriction site)**
pGPI-*gbnE*	5'-For: ATATCTAGAAGCAAGCGGTCCGACAGG (*Xba*I)
	5'-Rev: ATAAAGCTTATCGGCGTTGCCAAGCGA (*Hin*dIII)
	3'-For: TATAAGCTTTCGATGGCCGATTCGGGC (*Hin*dIII)
	3'-Rev: ATAGAATTCTGGCGGGTCGAGCCCATC (*Eco*RI)
	Screening-For: CTGTTGCAAGCGCACGATTAGC
	Screening-Rev: GACCAGGGGCAGTCGCAGCGA
pGPI-*gdsB_ER*	5'-For: *gatcccaagcttcttctaga*GCACTGTTCGAGAAGTAC
	5'-Rev: *gttgggtgaa*GTAGGCGTACTTGAGCTT
	3'-For: *gtacgcctac*TTCACCCAACTGCTCCG
	3'-Rev: *aacggctgacatgggaattc*GAAGATCGACGAGAG
	Screening-For: CAGGGCGTCGAGACATTCC
	Screening_Rev: CCTTGTCGCGCACGGATT
pMLBAD-*gbnE*	For: *tgggctagcaggaggaattc*ATGGCAATGATTACCGCA
	Rev: *tgggctagcaggaggaattc*ATGGCAATGATTACCGCA
pMLBAD_*etnL*	For: CCGGAATTCATGATTACCGCCAAAAGTCT (*Eco*RI)
(codon optimized)	Rev: CCCAAGCTTTTAACTGCTAACGCTCTGTG (*Hin*dIII)

Metabolite production, extraction, and UHPLC-ESI-Q-TOF-MS analysis

Burkholderia strains were grown on basal salts agar medium containing 0.4% (w/v) glycerol (BSM-G) at 30 °C for 72 h. For strains harboring pMLBAD-derived plasmids, 50 µg/ml trimethoprim and 0.2% (w/v) L-arabinose were added to the medium. Following incubation, the biomass was scraped off the plate, the agar was cut into 0.5 cm cubes and extracted twice using MeCN and the extract was passed through a 0.2 µm nylon centrifuge filter (Thermo Scientific). The filtered extract was analyzed using a Dionex UltiMate 3000 UHPLC connected to a Zorbax Eclipse Plus column (C18, 100 × 2.1 mm, 1.8 µm) coupled to a Bruker MaXis IMPACT ESI-Q-TOF mass spectrometer. The column was eluted with a gradient of 20% to 100% MeCN containing 0.1% formic acid at flow rate of 0.2 ml/min over 35 min. The mass spectrometer was operated in positive ion mode with a

scan range of 50-3000 *m/z*. Source conditions were: end plate offset at −500 V; capillary at −4500 V; nebulizer gas (N_2) at 1.6 bar; dry gas (N_2) at 8 L min^{-1}; dry temperature at 180 °C. Ion transfer conditions were: ion funnel RF at 200 Vpp; multiple RF at 200 Vpp; quadrupole low mass at 55 *m/z*; collision energy at 5.0 eV; collision RF at 600 Vpp; ion cooler RF at 50–350 Vpp; transfer time at 121 s; pre-pulse storage time at 1 s. Calibration was performed with 1 mM sodium formate through a loop injection of 20 µL at the start of each run.

Synthesis of (2*E*, 4*E*)-2,4-hexadienoyl NAC thioester 7 and pantetheine thioester 17

(2E, 4E)-2,4-hexadienoyl NAC thioester 7

(2*E*, 4*E*)-2,4-hexadienoyl NAC thioester **7** was synthesized using the same procedure as that used for the synthesis of (*E*)-4-hexenoyl NAC thioester **19** (see below) except that sorbic acid (100 mg, 0.89 mmol) was used in place of (*E*)-4-hexenoic acid and the scale of the reaction was adjusted accordingly. This afforded the desired product as a white solid (176 mg, 93 %).

ν_{max}/cm^{-1} (neat) 3327 (NH), 1665, 1532 (C=O); δ_H (500 MHz; $CDCl_3$) 7.20 (1H, dd, J 15.0 and 10.5, C*H*CHCOS), 6.25 (1H, dq, J 15.0 and 7.0, CH_3C*H*), 6.16 (1H, ddd, J 15.0, 11.0 and 1.0, CH_3CHC*H*), 6.07 (1H, d, J 15.0, C*H*COS), 5.91 (1H, m, N*H*), 3.45 (2H, q, J 6.0, CH_2NH), 3.07 (2H, t, J 6.5, CH_2S), 1.96 (3H, s, COCH_3), 1.85 (3H, d, J 6.5, CHCH_3); δ_C (125 MHz, $CDCl_3$) 191.6 (*C*OS), 170.3 (*C*ON), 142.1 (CH_3*C*H), 142.0 (*C*HCHCOS), 129.7 (CH_3CH*C*H), 125.8 (CH*C*HCOS), 40.0 **(**CH_2NH**)**, 28.5 (CH_2S), 23.4 (COCH_3), 14.6 (CHCH_3); HRMS (ESI) cald. for $C_{10}H_{15}NNaO_2S$ (M + Na^+) requires 236.0721, found 236.0720.

(2E, 4E)-2,4-hexadienoyl pantetheine thioester 17

(2E, 4*E)*-2,4-hexadienoyl pantetheine thioester **17** was synthesized as described previously.[5]

Synthesis of (*E*)-4-hexenoyl pantetheine thioester 18 and NAC thioester 19

***(E)-4-hexenoyl pantetheine thioester* 18**

(*R*)-*S*-(2-(3-(2,2,5,5-tetramethyl-1,3-dioxane-4-carboxamido)propanamido)ethyl) (*E*)-hex-4-enethioate was synthesized using the same procedure as that used for the synthesis of (*E*)-4-hexenoyl NAC thioester **19** (see below) except that (*R*)-*N*-(3-((2-mercaptoethyl)amino)-3-oxopropyl)-2,2,5,5-tetramethyl-1,3-dioxane-4-carboxamide[5] (134 mg, 0.42 mmol, 1.4 equiv.) was used in place of *N*-(2-mercaptoethyl)acetamide and the scale of the reaction was adjusted accordingly. This afforded *(R)*-*S*-(2-(3-(2,2,5,5-tetramethyl-1,3-dioxane-4-carboxamido)propanamido)ethyl) *(E)*-hex-4-enethioate as a colorless oil (123 mg, 76 %).

ν_{max}/cm^{-1} (neat) 3338 (NH), 1665, 1538 (C=O); δ_H (500 MHz; $CDCl_3$) 7.02 (1H, br. t, J 6.0, N*H*), 6.16 (1H, br. t,

J 5.5, N*H*CH_2CH_2S), 5.48 (1H, dq, J 15.5 and 6.5, C*H*CH_3), 5.37 (1H, dt, J 15.5 and 6.5, C*H*$CHCH_3$), 4.07 (1H, s, C*H*CONH), 3.67 (1H, d J 11.5, $CH_2OC(CH_3)_2$), 3.60-3.35 (4H, m, $NHCH_2$, CH_2CH_2S), 3.27 (1H, d J 12.0, $CH_2OC(CH_3)_2$), 3.00 (2H, t, J 6.5, CH_2S), 2.61 (2H, t, J 7.5, CH_2COS), 2.41 (2H, t, J 6.0, CH_2CONH), 2.32 (2H, q, J 7.0, CH_2CH_2COS), 1.63 (3H, d, J 6.0, $CHCH_3$), 1.45 (3H, s, $OC(CH_3)_2$), 1.41 (3H, s, $OC(CH_3)_2$), 1.03 (3H, s, $CH_2C(CH_3)_2$), 0.96 (3H, s, $CH_2C(CH_3)_2$); δ_C (125 MHz, $CDCl_3$) 199.3 (CO_2S), 171.3 (CH_2CONH), 170.2 (CHCONH), 128.6 (CH_3CHCH), 126.8 (CH_3CH), 99.2 ($OC(CH_3)_2$), 77.3 (CH), 71.6 ($CH_2OC(CH_3)_2$), 44.1 (CH_2COS), 39.7 (CH_2CH_2S), 36.1 (CH_2CONH), 34.9 (CH_2NH), 33.1 ($CH_2C(CH_3)_2$), 29.6 ($OC(CH_3)_2$), 28.6 **(**CH_2CH_2COS**),** 28.5 (CH_2S), 22.3 ($CH_2C(CH_3)_2$), 19.0 ($OC(CH_3)_2$), 18.8 ($CH_2C(CH_3)_2$), 18.0 ($CHCH_3$); HRMS (ESI) cald. for $C_{20}H_{34}N_2NaO_5S$: 437.2086, found 437.2085 ($[M+Na]^+$); $[\alpha]_D^{27}$ (c 0.1, $CHCl_3$): +25.3.

(R)-S-(2-(3-(2,2,5,5-tetramethyl-1,3-dioxane-4-carboxamido)propanamido)ethyl) *(E)*-hex-4-enethioate (70 mg, 0.17 mmol, 1.0 equiv.) was stirred in AcOH : H_2O (2 : 1, 3 mL), for 16 h at room temperature. The mixture was concentrated *in vacuo* and purified using silica chromatography (CH_2Cl_2 / MeOH, 9:1) to give the product as a colorless oil (52 mg, 82 %).

ν_{max}/cm^{-1} (neat) 3357 (OH), 3301 (NH), 1657, 1541 (C=O); δ_H (500 MHz; CD_3OD) 5.49 (1H, dq, J 15.5 and 6.0, C*H*CH_3), 5.41 (1H, dt, J 15.5 and 6.5, C*H*$CHCH_3$), 3.89 (1H, s, C*H*CONH), 3.53-3.37 (6H, m, $NHCH_2$, CH_2CH_2S, CH_2OH), 2.99 (2H, t, J 6.5, CH_2S), 2.62 (2H, t, J 7.5, CH_2COS), 2.40 (2H, t, J 6.5, CH_2CONH), 2.30 (2H, q, J 7.0, CH_2CH_2COS), 1.63 (3H, d, J 6.5, $CHCH_3$), 0.92 (6H, s, $C(CH_3)_2$); δ_C (125 MHz, CD_3OD) 200.0 (CO_2S), 176.1 (CH_2CONH), 173.9 (CHCONH), 130.0 (CH_3CHCH), 127.5 (CH_3CH), 77.3 (CH), 70.4 (CH_2OH), 44.8 (CH_2COS), 40.4 ($CH_2C(CH_3)_2$), 40.1 (CH_2CH_2S), 36.4 (CH_2CONH), 36.3 (CH_2NH), 29.5 (CH_2CH_2COS), 29.1 (CH_2S), 21.3 ($CH_2C(CH_3)_2$), 20.9 ($CH_2C(CH_3)_2$), 18.0 ($CHCH_3$); HRMS (ESI) cald. for $C_{17}H_{30}N_2NaO_5S$: 397.1773, found 397.1770 ($[M+Na]^+$); $[\alpha]_D^{27}$ (c 0.2, MeOH): +46.3.

(*E*)-4-hexenoyl NAC thioester 19

To a solution of (*E*)-4-hexenoic acid (76 mg, 0.67 mmol, 1.3 equiv.),[6] *N*-(2-mercaptoethyl)acetamide (88 mg, 0.74 mmol, 1.4 equiv.) and DMAP (18 mg, 0.16 mmol, 0.3 equiv.) in CH_2Cl_2 (5 mL), was added EDC (141 mg, 0.75 mmol, 1.4 equiv.) at 0 ^oC. The resulting mixture was stirred at room temperature overnight, quenched by the addition of 2 M HCl, extracted with CH_2Cl_2 (3 x 10 mL), washed with brine (10 mL), dried ($MgSO_4$), filtered and concentrated *in vacuo*. Purification using silica gel chromatography (EtOAc) afforded the desired product as a colorless oil (131 mg, 91 %).

ν_{max}/cm^{-1} (neat) 3326 (NH), 1659, 1545 (C=O); δ_H (500 MHz; $CDCl_3$) 5.80 (1H, br. s, N*H*), 5.49 (1H, dq, J 15.0 and 6.5, CH_3CH), 5.38 (1H, dt, J 15.5 and 6.5, CH_3CHCH), 3.43 (2H, q, J 6.0, CH_2NH), 3.02 (2H, t, J 6.5,

CH_2S), 2.63 (2H, t, J 7.5, CH_2COS), 2.34 (2H, q, J 7.0, CH_2CH$_2$COS), 1.96 (3H, s, COCH_3), 1.64 (3H, d, J 6.5, CHCH_3); δ_C (125 MHz, CDCl$_3$) 199.8 (*C*OS), 170.4 (*C*ON), 128.6 (CH$_3$CH*C*H), 126.9 (CH$_3$*C*H), 44.1 (*C*H$_2$COS), 39.9 **(***C*H$_2$NH**),** 28.6 **(***C*H$_2$CH$_2$COS**),** 28.6 (*C*H$_2$S), 23.4 (CO*C*H$_3$), 18.0 (CH*C*H$_3$); HRMS (ESI) cald. for $C_{10}H_{17}NNaO_2S$ ([M+Na]$^+$) requires 238.0878, found 238.0880.

Figures

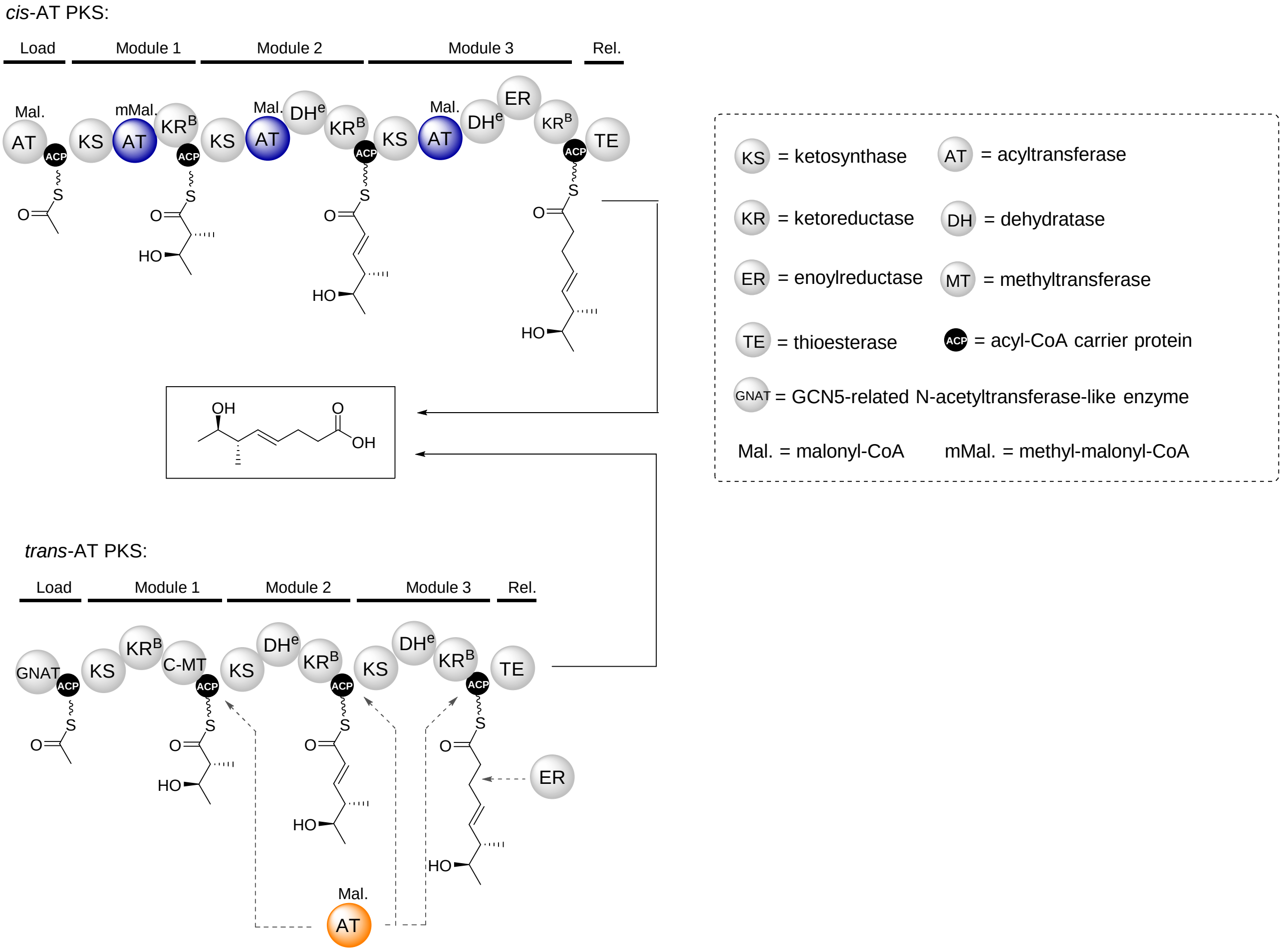

Figure S1. Comparison of *cis* and *trans*-AT PKS architectures. (A) *Cis*-AT PKSs have AT domains (in blue) integrated into each module that load a specific (alkyl)malonyl extender unit onto the downstream ACP domain, (B) *Trans*-AT PKSs typically employ a single AT (in orange) to load a malonyl extender unit onto the ACP domain in each module. *Trans*-AT PKSs usually employ other *trans*-acting enzymes (such as ERs) to modify the growing chain. Subtypes of KR and DH domains are indicated as KR^B (type B KR domain) and DH^e (2-enoyl thioester forming DH domain). β-Keto-thioester intermediates resulting from chain elongation catalyzed by KS domains are reduced by type B and type A KR domains to (3*R*)-3-hydroxy and (3*S*)-3-hydroxy-thioesters, respectively, followed by DH^e domain-catalyzed removal of the *pro*-S hydrogen atom at C-2 and elimination of the C-3 hydroxyl group, forming *E* or *Z*-enoyl thioesters. Other subtypes of DH domains exist: 2, 4-dienoyl thioester synthases (DH^d domains); 2-enoyl thioester isomerases (DH^i domains) and tetrahydropyran/tetrahydrofuran synthases, (DH^t domains).[5]

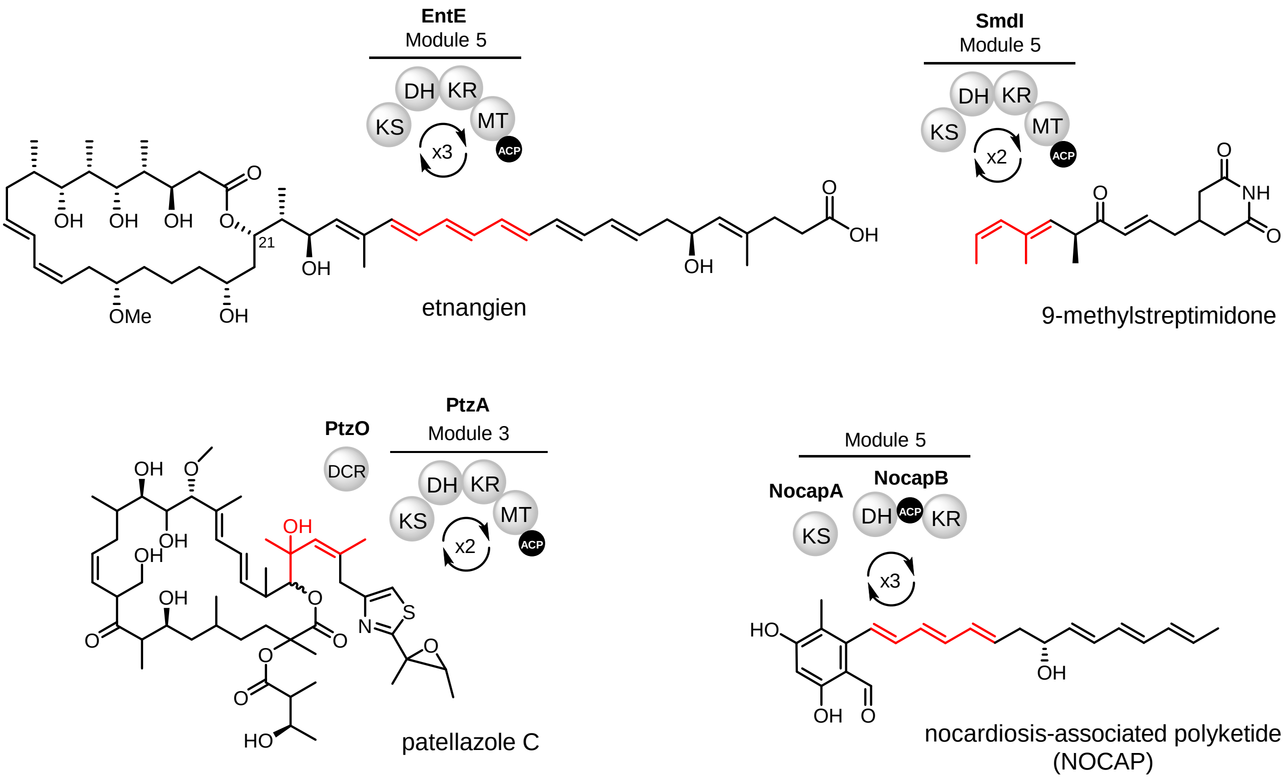

Figure S2. Examples of polyketides assembled by *trans*-AT PKSs containing putative intrinsically iterative modules. Moieties derived from module iteration are highlighted in red.

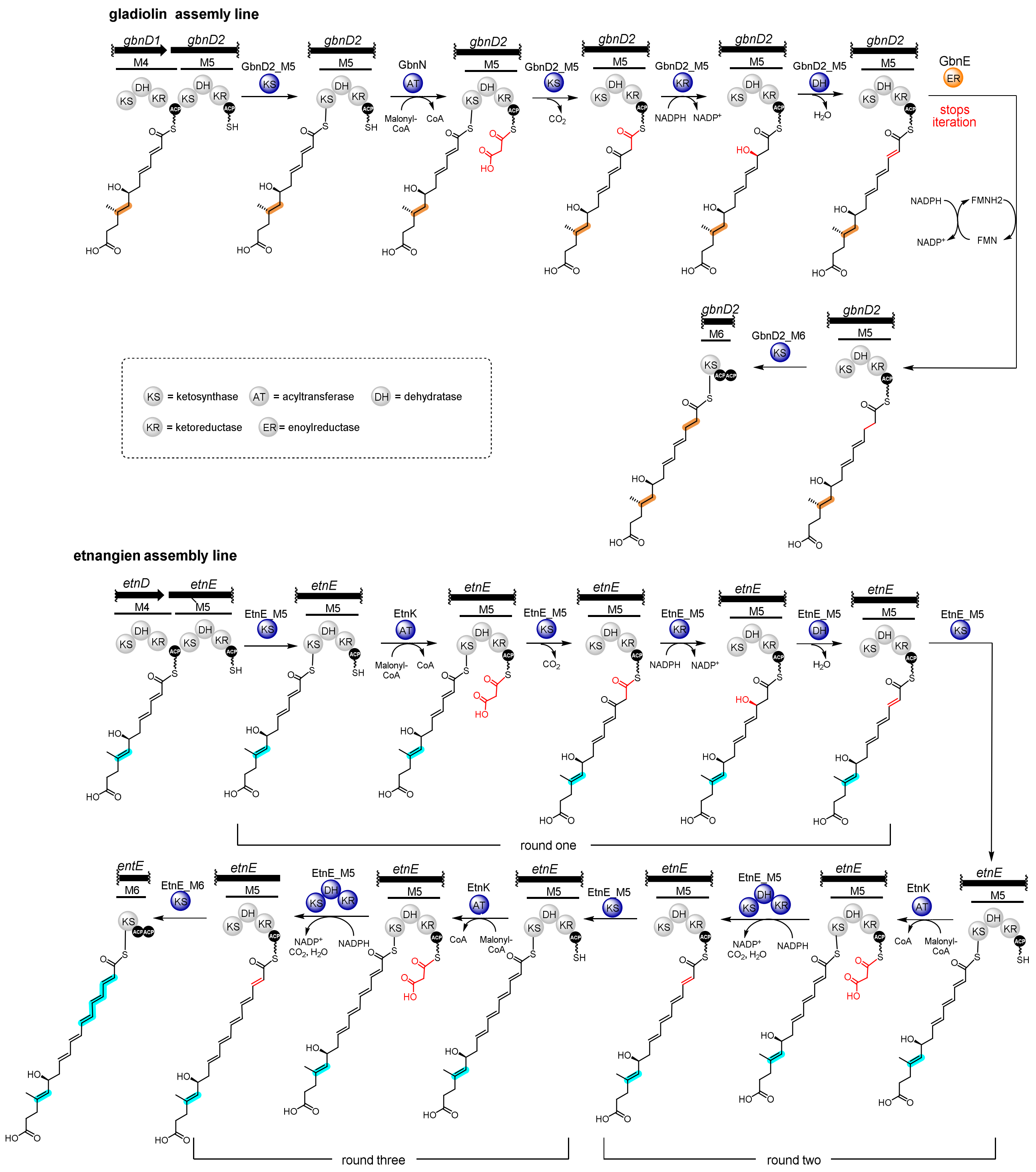

Figure S3. Comparison of transformations proposed to be catalyzed by module 5 of the gladiolin and etnangien PKSs. After receiving the nascent polyketide chain from module 4, module 5 of the gladiolin PKS catalyzes one round of chain elongation, ketoreduction and dehydration, followed by enoyl reduction catalyzed by the *trans*-acting ER GbnE, and transfer of the product to module 6. In the etnangien PKS, following transfer of the nascent polyketide from module 4 to module 5, the latter catalyzes three rounds of chain elongation, ketoreduction and dehydration, without the intervention of a *trans*-acting ER, after which it is transferred to module 6. Structural changes in each intermediate are highlighted in red. Structural differences between gladiolin and etnangien biosynthetic intermediates are highlighted in orange and blue, respectively.

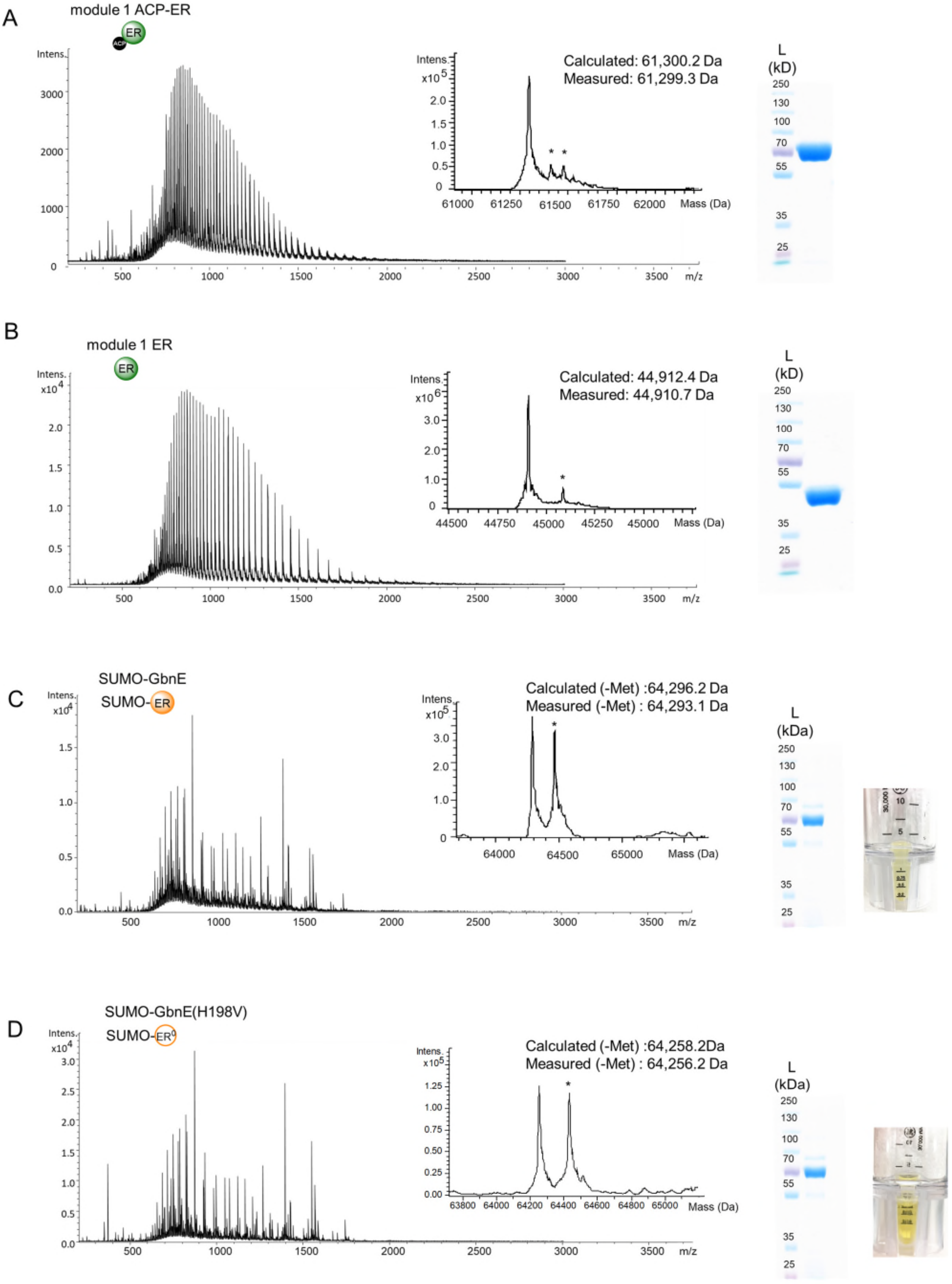
A
module 1 ACP-ER
ER
Calculated: 61,300.2 Da
Measured: 61,299.3 Da
Mass (Da)
m/z
L
(kD)
B
module 1 ER
ER
Calculated: 44,912.4 Da
Measured: 44,910.7 Da
Mass (Da)
m/z
L
(kD)
C
SUMO-GbnE
SUMO-ER
Calculated (-Met) :64,296.2 Da
Measured (-Met) : 64,293.1 Da
Mass (Da)
m/z
L
(kDa)
D
SUMO-GbnE(H198V)
SUMO-ER0
Calculated (-Met) :64,258.2Da
Measured (-Met) : 64,256.2 Da
Mass (Da)
m/z
L
(kDa)

E

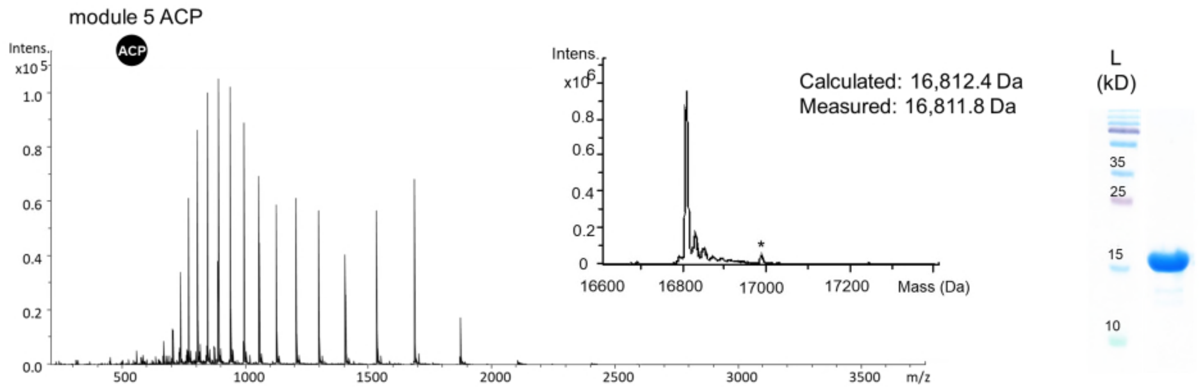

module 5 ACP
ACP
Intens.
x10 5
m/z
Intens.
x10 6
Mass (Da)
Calculated: 16,812.4 Da
Measured: 16,811.8 Da
*
L
(kD)
35
25
15
10

F

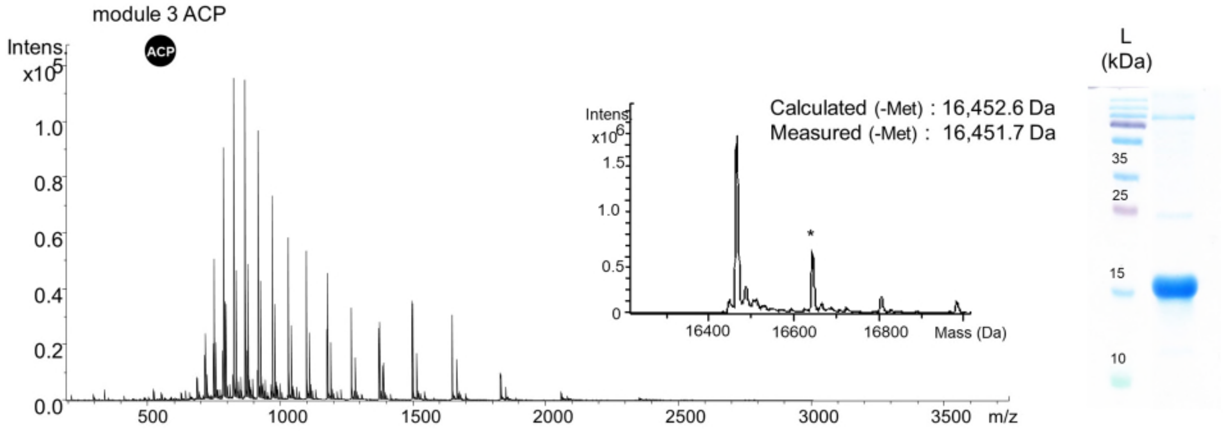

module 3 ACP
ACP
Intens.
x10 5
m/z
Intens.
x10 6
Mass (Da)
Calculated (-Met) : 16,452.6 Da
Measured (-Met) : 16,451.7 Da
*
L
(kDa)
35
25
15
10

G

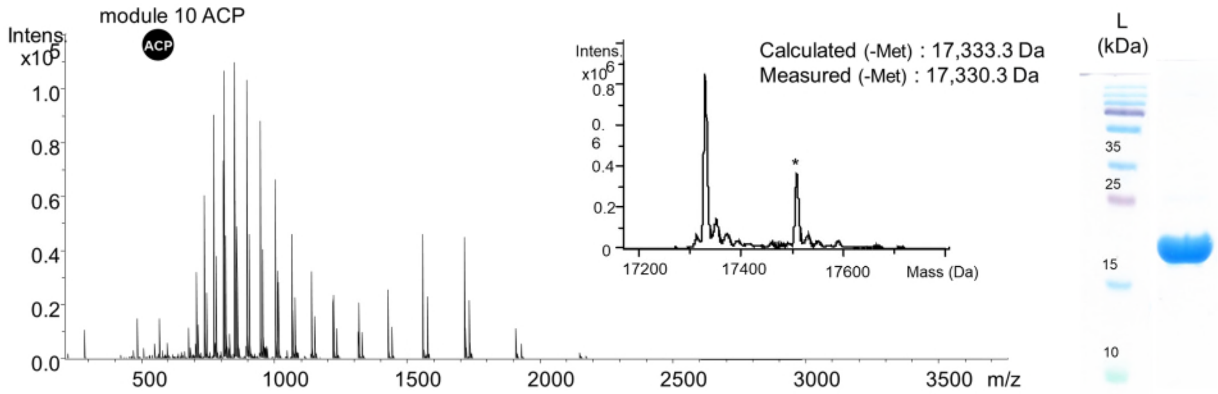

module 10 ACP
ACP
Intens.
x10 5
m/z
Intens.
x10 6
Mass (Da)
Calculated (-Met) : 17,333.3 Da
Measured (-Met) : 17,330.3 Da
*
L
(kDa)
35
25
15
10

H

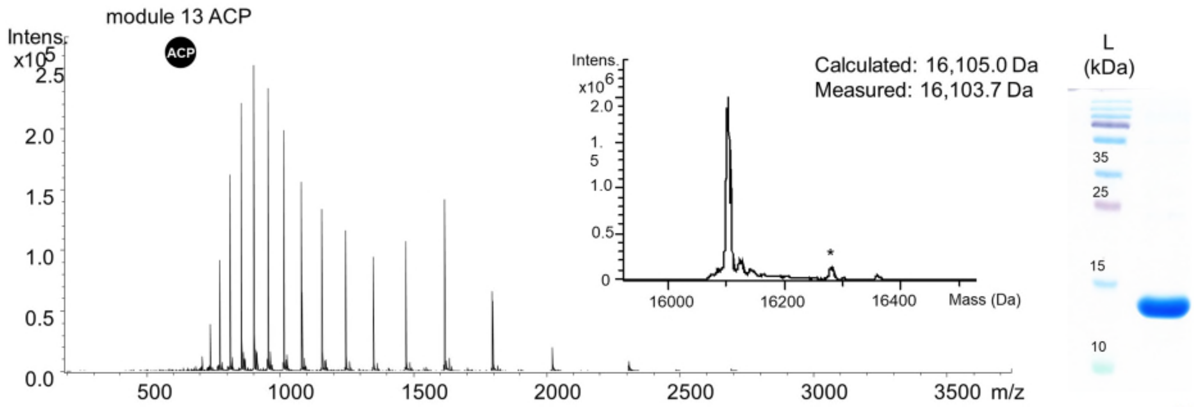

module 13 ACP
ACP
Intens.
x10 5
m/z
Intens.
x10 6
Mass (Da)
Calculated: 16,105.0 Da
Measured: 16,103.7 Da
*
L
(kDa)
35
25
15
10

I

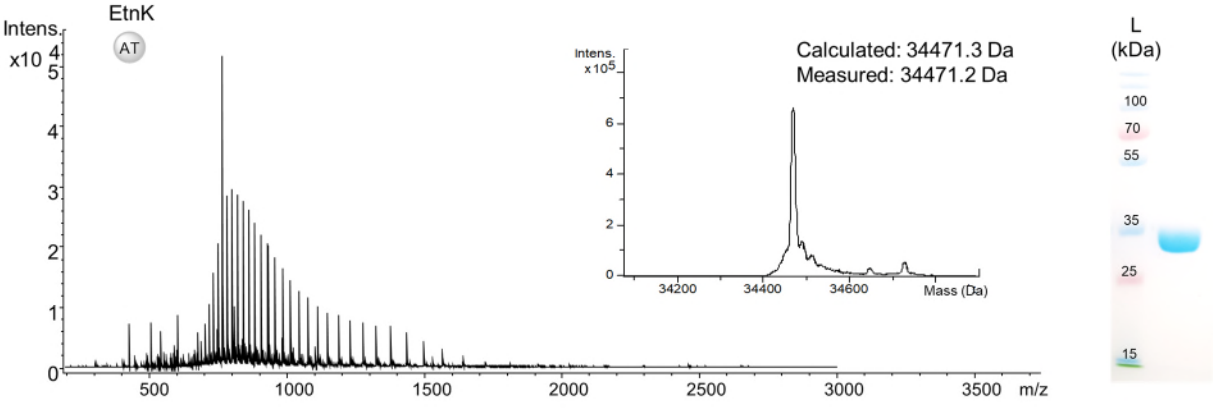
EtnK
AT
Calculated: 34471.3 Da
Measured: 34471.2 Da
Intens. x10 4
m/z
Mass (Da)
L (kDa)

J

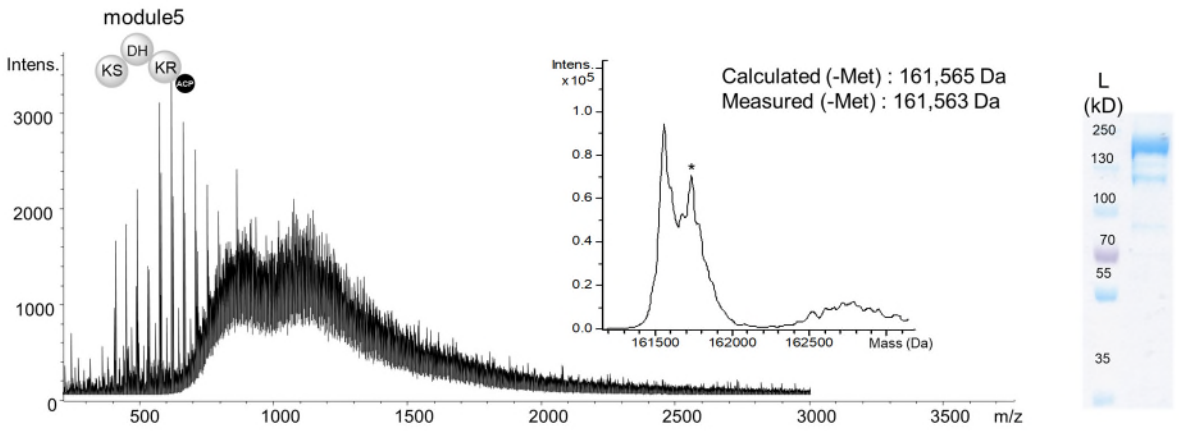
module5
KS
DH
KR
ACP
Calculated (-Met) : 161,565 Da
Measured (-Met) : 161,563 Da
Intens.
m/z
Mass (Da)
L (kD)

K

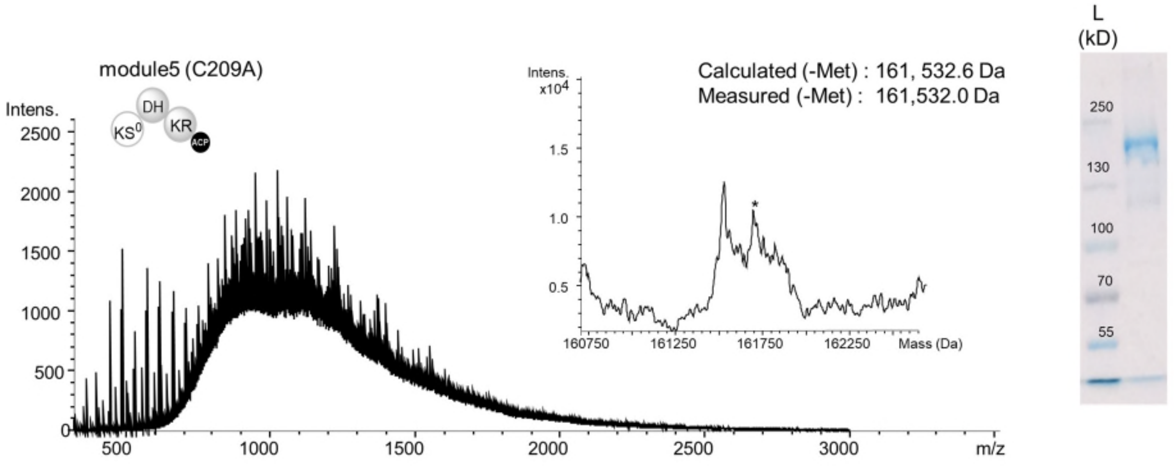
module5 (C209A)
KS0
DH
KR
ACP
Calculated (-Met) : 161, 532.6 Da
Measured (-Met) : 161,532.0 Da
Intens.
m/z
Mass (Da)
L (kD)

L

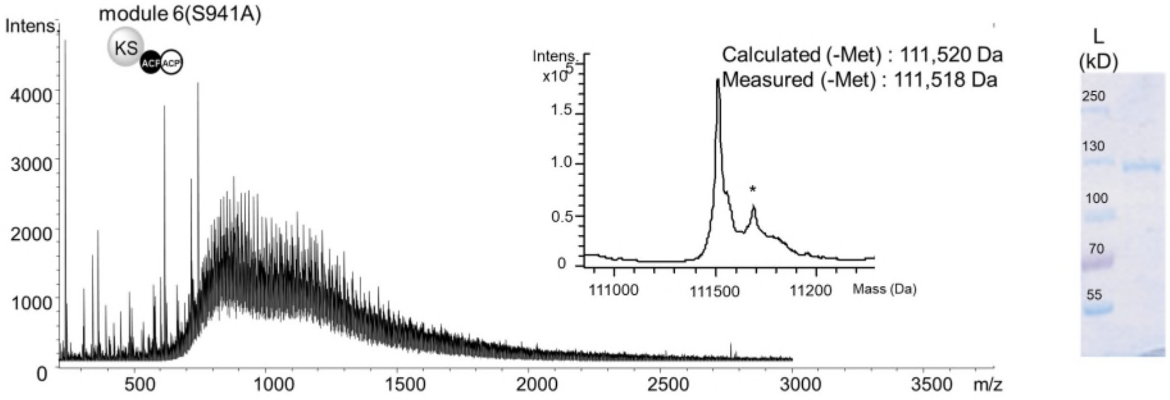
module 6(S941A)
KS
ACP
ACP
Calculated (-Met) : 111,520 Da
Measured (-Met) : 111,518 Da
Intens.
m/z
Mass (Da)
L (kD)

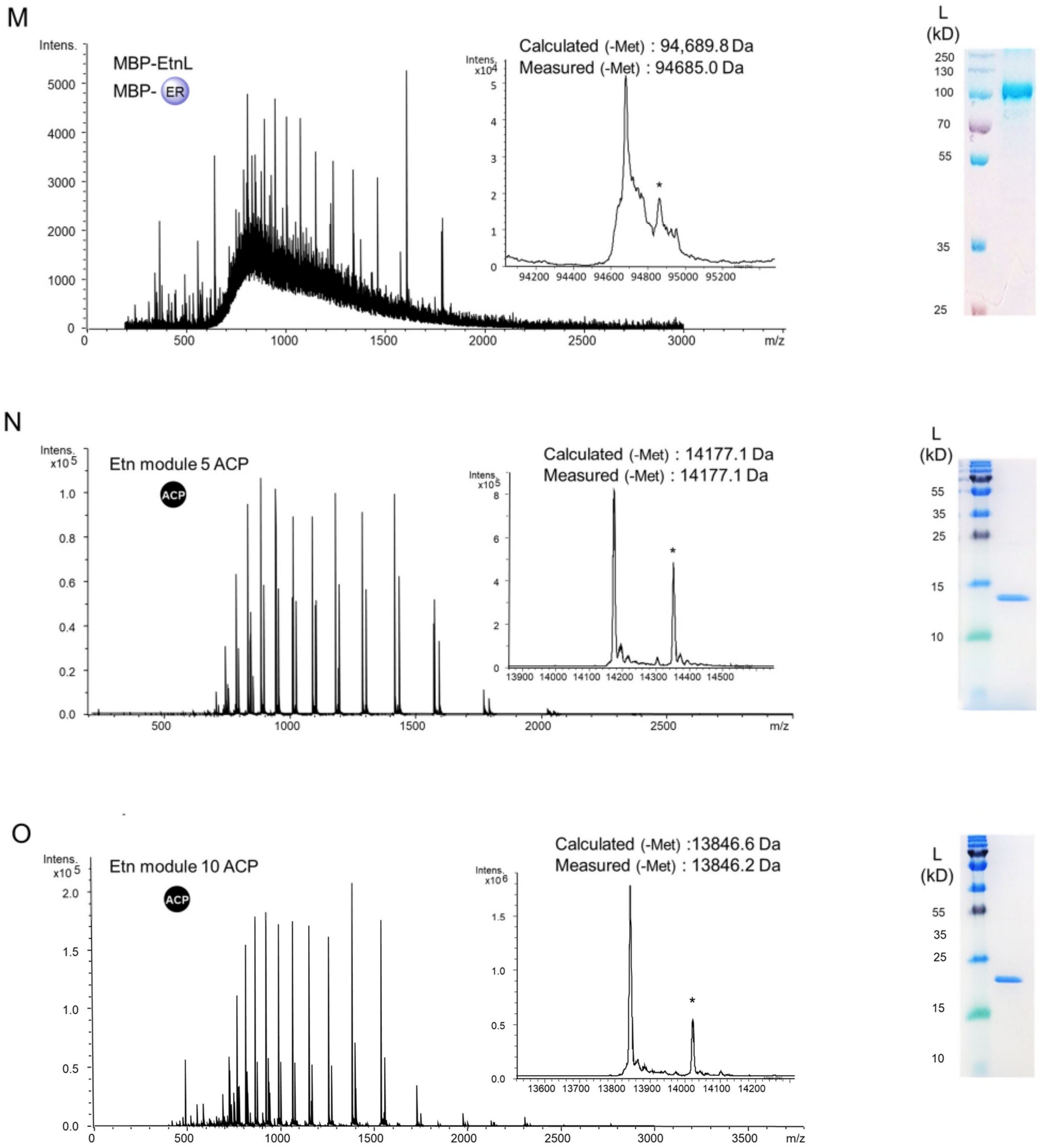

Figure S4. UHPLC-ESI-Q-TOF-MS and SDS-PAGE characterization of purified recombinant proteins. Raw mass spectra (left), deconvoluted mass spectra (middle) and SDS-PAGE gels (right) of purified recombinant proteins. Peaks labeled with '*' correspond to a gluconylated form of the protein resulting in a mass increase of 178 Da due to gluconylation of the His-tag.[7] ACP domains from which the partially gluconylated His-tags have been cleave behaved identically in enzymatic assays, confirming gluconylation doesn't affect their structure or function. Purified SUMO-GbnE and SUMO-GbnE-H198V were yellow, consistent with a bound flavin cofactor. Purified MBP-EtnL did not show an obvious yellow color, but turnover in enoyl reduction assays using MBP-EntL without addition of FMN indicates MBP-EntL is purified with the flavin cofactor bound. The N-terminal Met residue of some proteins was removed by an internal aminopeptidase during overproduction in *E. coli* BL21 (DE3),[8] indicated as '(-Met)' in the calculated and measured masses.

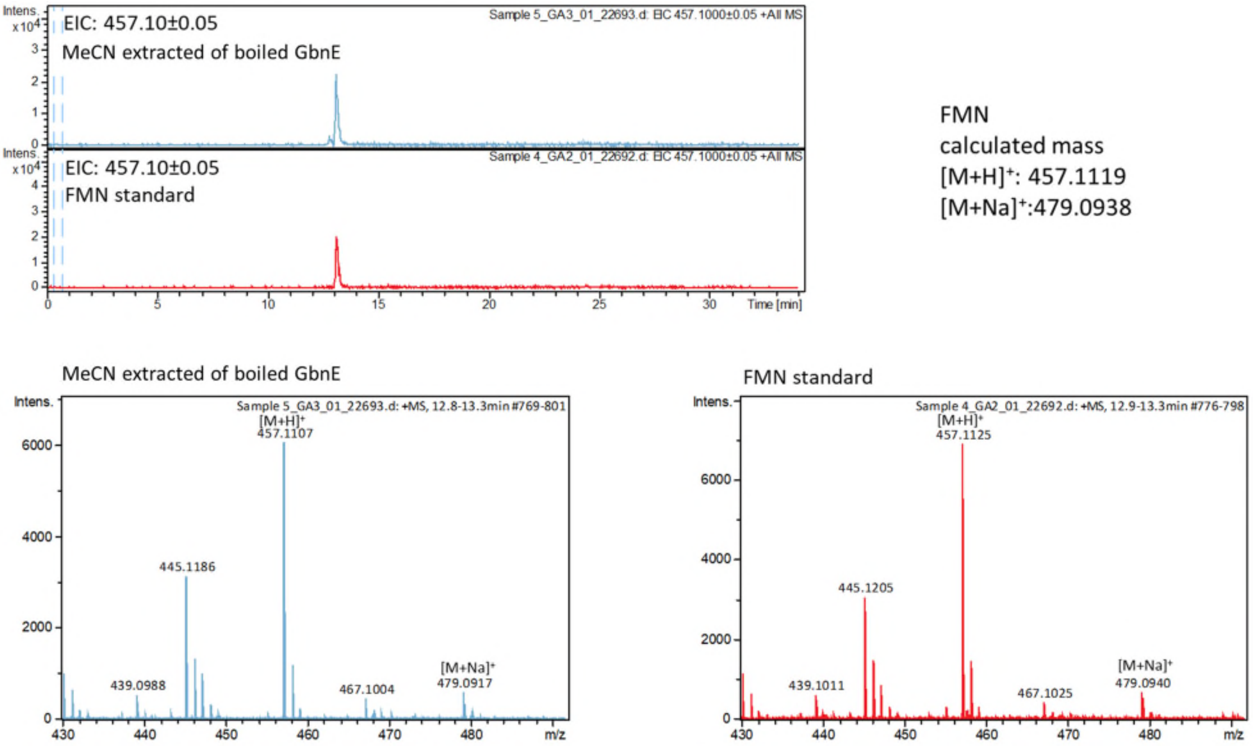

Figure S5. UHPLC-ESI-Q-TOF-MS analysis of an MeCN extract of GbnE confirming it purifies with FMN bound. The extracted ion chromatogram (top panel) corresponding to $[M+H]^+$ for FMN (*m/z* = 457.10± 0.05) from an MeCN extract of boiled GbnE (top chromatogram) contained a species with the same retention time as that for an authentic standard of FMN (bottom chromatogram). High resolution mass spectra of FMN in the MeCN extract of GbnE (bottom left panel) and an authentic standard of FMN (bottom right panel). The *m/z* values observed for the $[M+H]^+$ and $[M+Na]^+$ ions are in good agreement with the calculated values (top right).

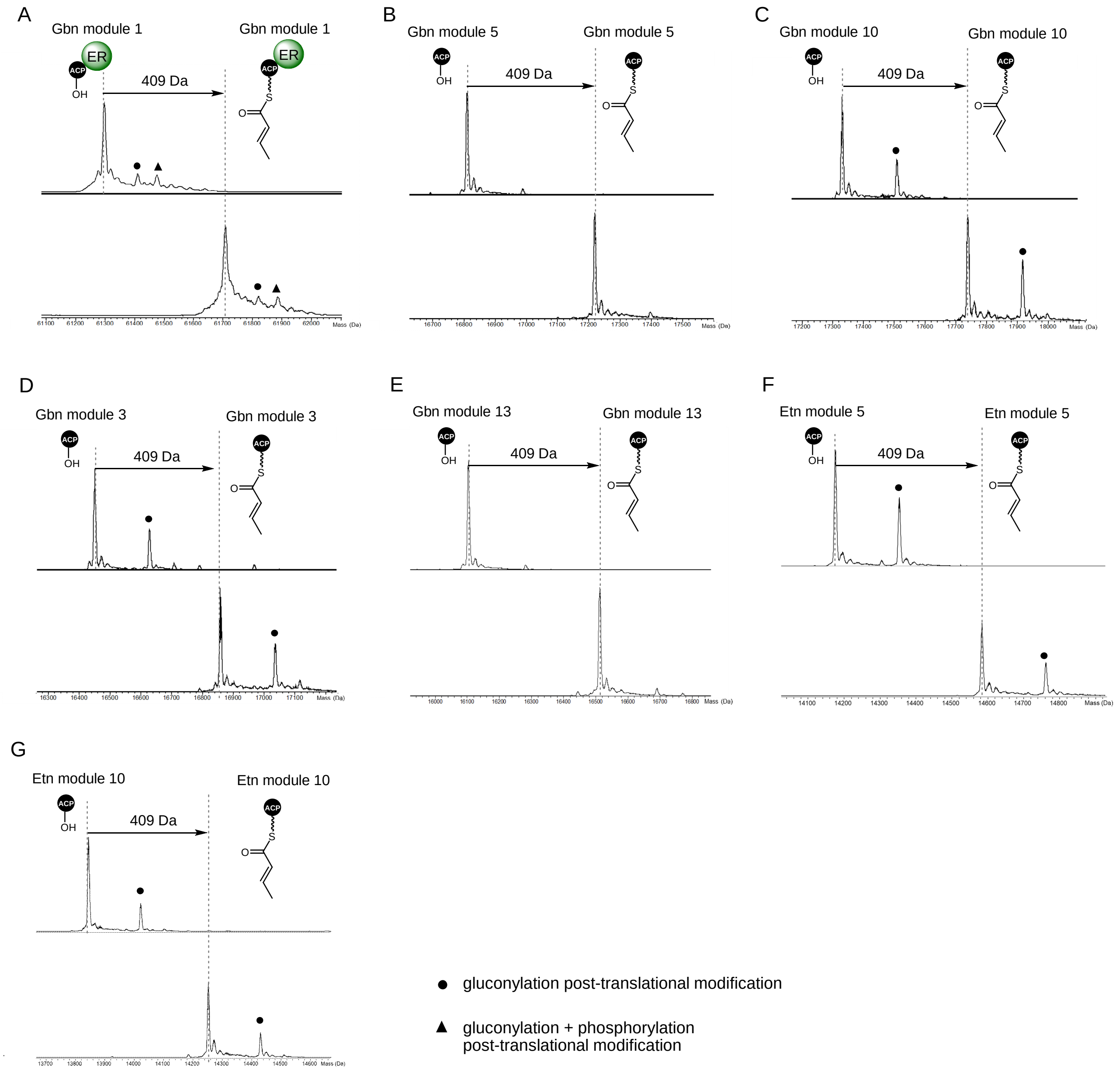

Figure S6. Deconvoluted mass spectra from UHPLC-ESI-Q-TOF-MS analysis of *apo*-ACP domain conversion to crotonylated *holo* forms. Gluconylated and gluconylated + phosphorylated species are indicated with a dot and triangle, respectively. A mass shift of 409 Da is observed for conversion from the *apo* to crotonylated *holo* form.

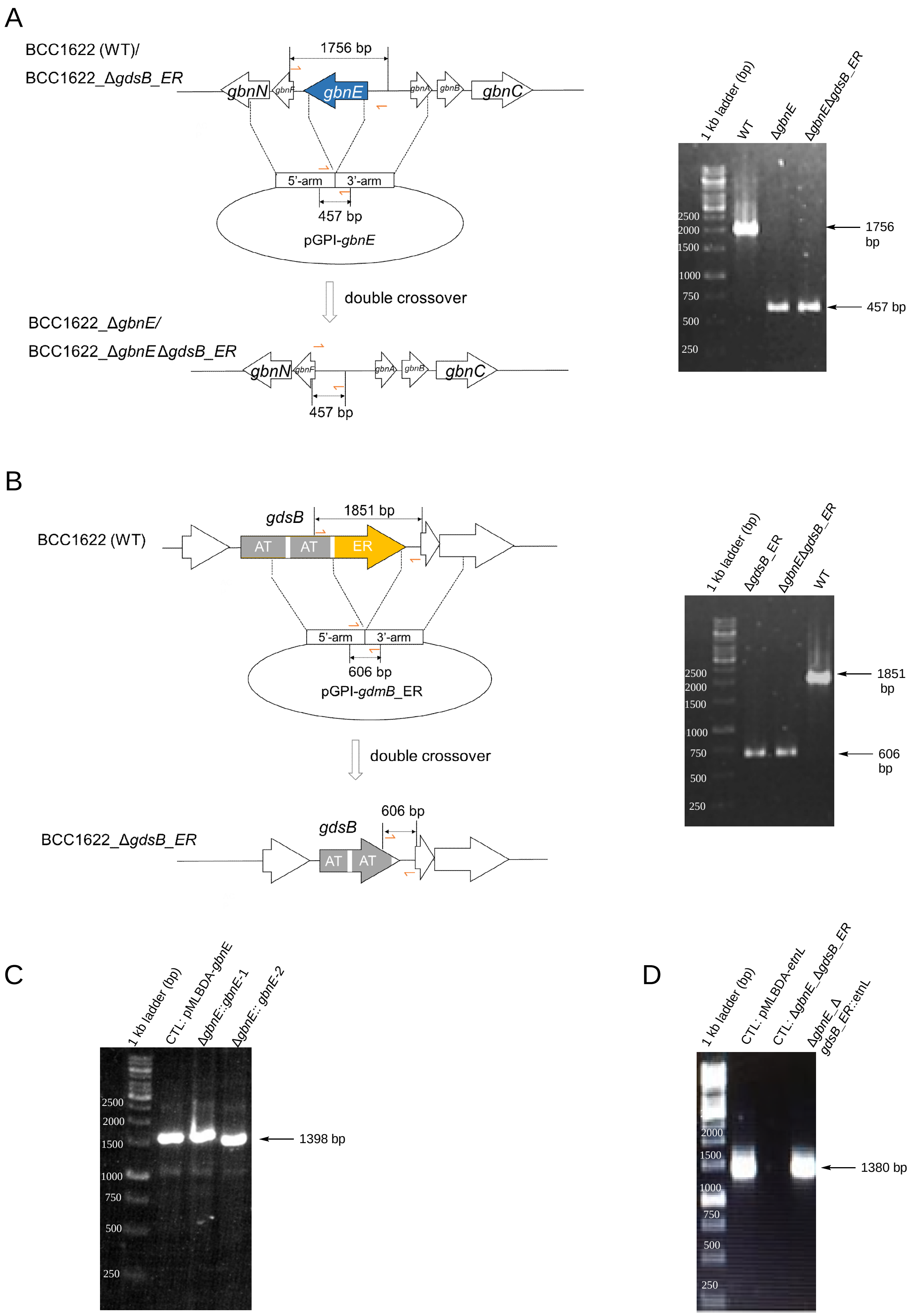

Figure S7. Construction of in-frame deletion mutants in *B. gladioli* BCC1622. (A) Scheme for construction of *gbnE* in-frame deletion and PCR confirmation of the expected genotype. (B) Scheme for construction of *gdsB*_ER in-frame deletions and PCR confirmation of the expected genotype. (C) PCR confirmation of the genotype of the Δ*gbnE*::*gbnE* mutant. (D) PCR confirmation of the genotype of the Δ*gdsB*_ERΔ*gbnE*::*etnL* mutant.

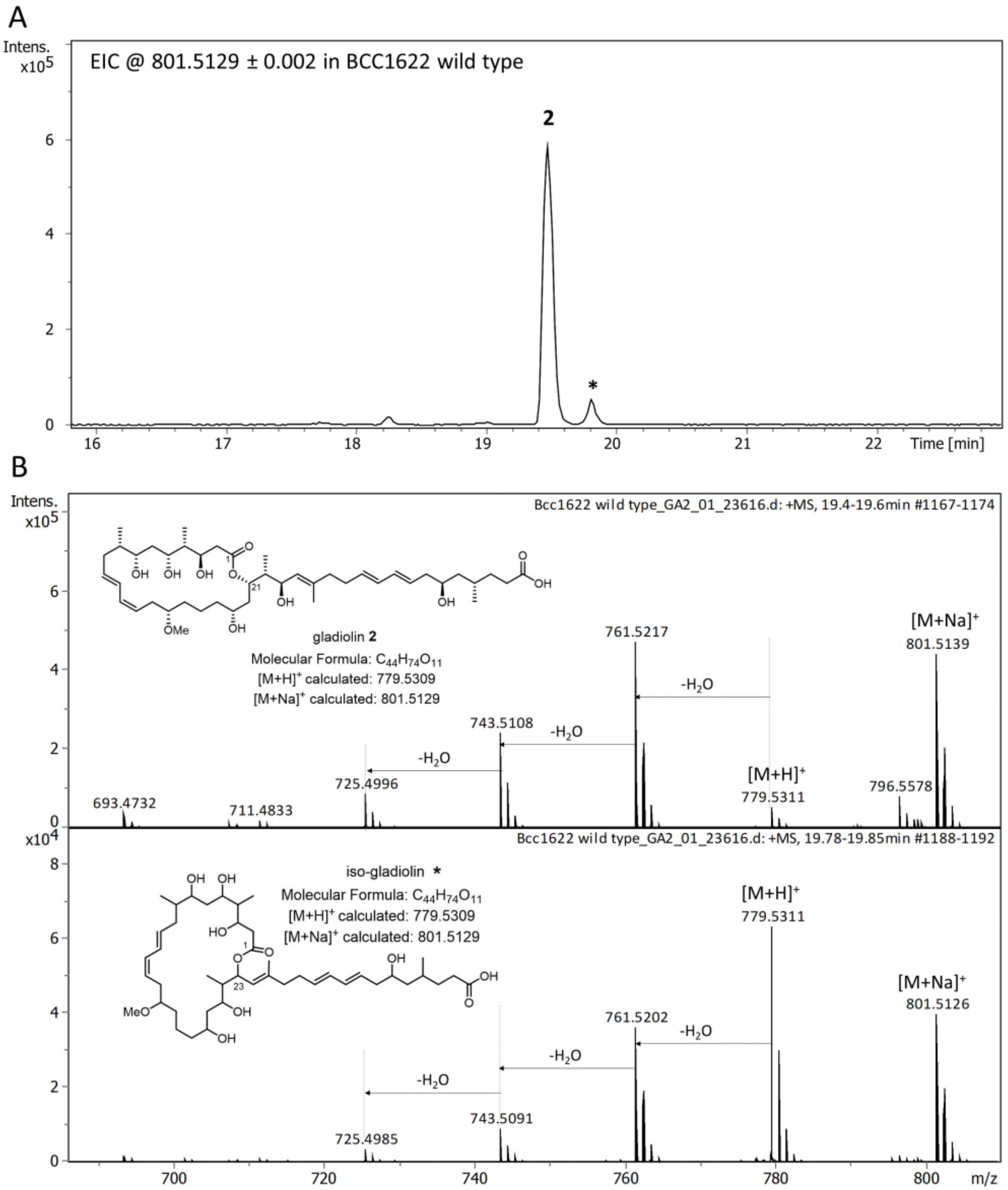

Figure S8. UHPLC-ESI-Q-TOF-MS analysis of gladiolin and *iso*-gladiolin in extracts of *B. gladioli* BCC1622. (A) Extracted ion chromatogram corresponding to $[M+Na]^+$ for gladiolin **2** and *iso*-gladiolin (*****) (*m/z* = 801.5129 ± 0.002) from analysis of *B. gladioli* BCC1622 organic extracts. (B) High resolution mass spectra of gladiolin **2** and iso-gladiolin (*****). The observed *m/z* values for the $[M+H]^+$ and $[M+Na]^+$ ions are in excellent agreement with the calculated values.

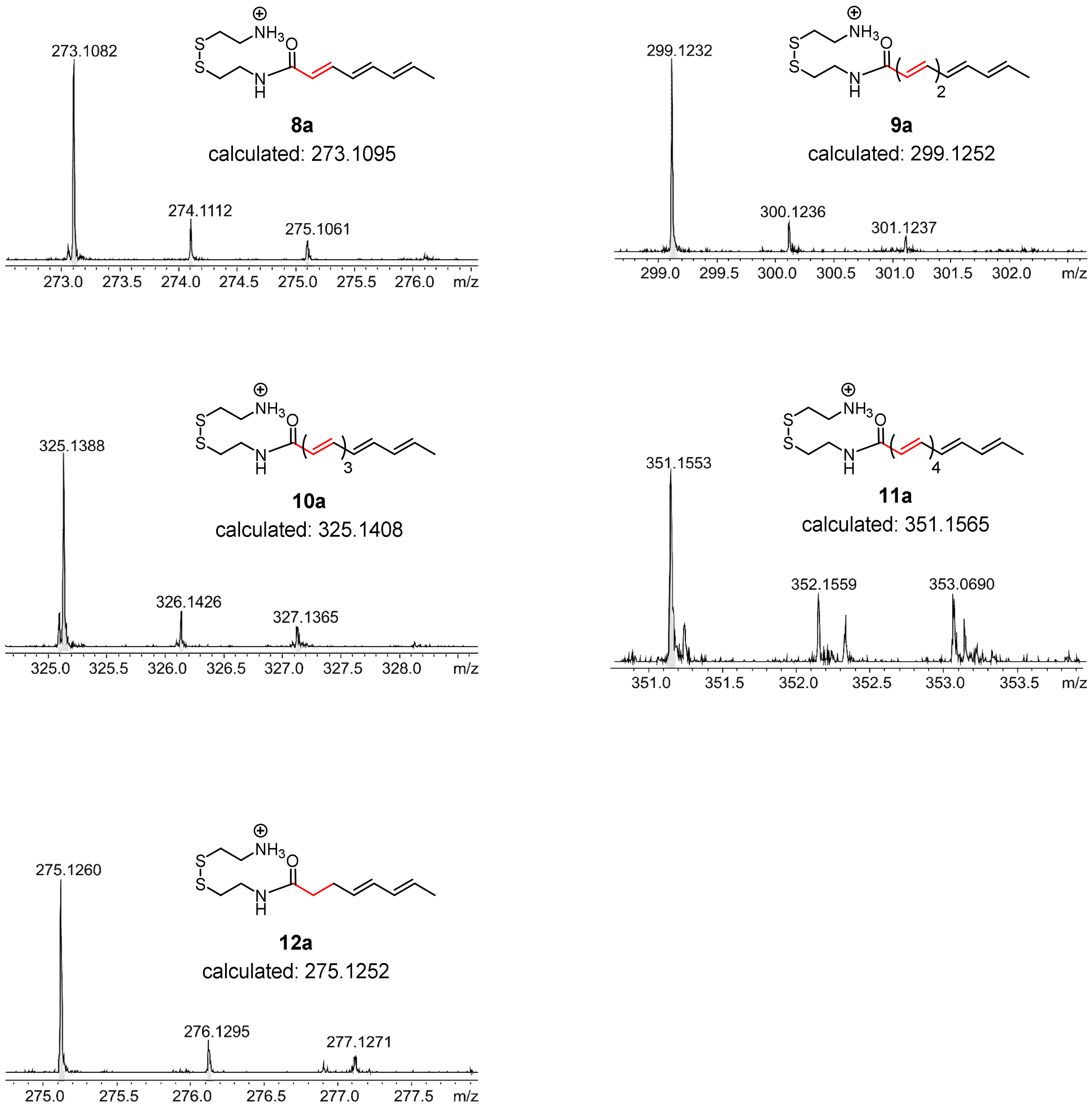

Figure S9. High resolution mass spectra of cysteamine cleavage adducts observed in UHPLC-ESI-Q-TOF-MS analyses of module 5 *in vitro* reconstitution assays in the presence and absence of GbnE or an H198V mutant. Representative spectra of the $[M+H]^+$ ions for **8a**, **9a**, **10a**, **11a** and **12a** and comparison with the calculated *m/z* for each species.

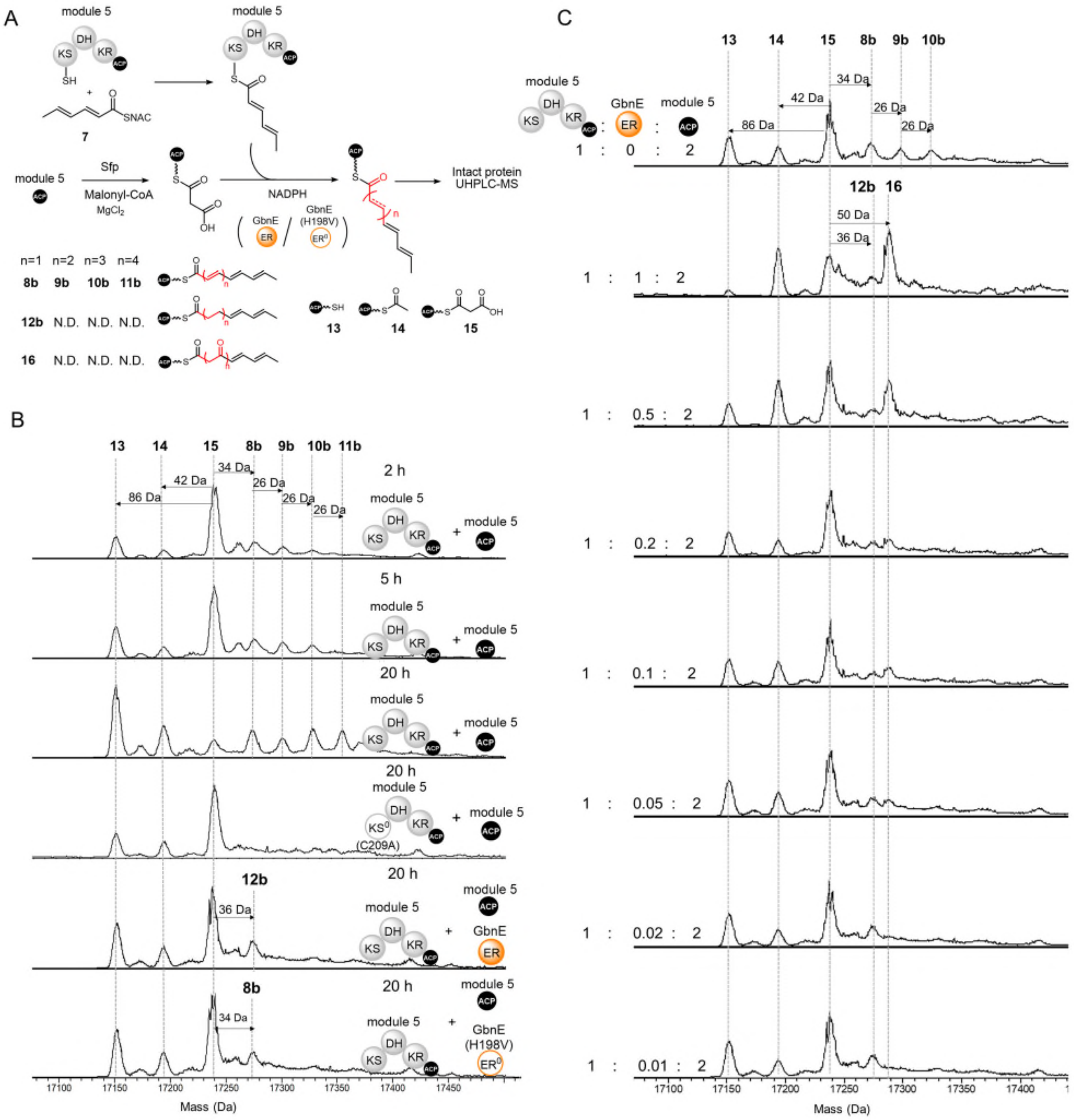

Figure S10. Deconvoluted mass spectra from UHPLC-ESI-Q-TOF-MS analysis of the module 5 *in vitro* reconstitution in the presence of the excised module 5 ACP domain and presence/absence of GbnE/GbnE(H198V). (A) Scheme illustrating the assay design. (B) Spectra of the excised module 5 ACP domain from the *in vitro* reconstitution assays in the absence of GbnE after 2 h (top), 5 h (second from top) and 20 h (third from top) and in the presence of GbnE after 20 h (second from bottom) and the presence of GbnE(H198V) after 20 h (bottom). The spectrum of the excised module 5 ACP domain from a control reaction in which module 5 was replaced by a C209A mutant with an inactive KS domain is third from bottom. (C) Spectra of the excised module 5 ACP domain from the *in vitro* reconstitution assays containing varying amounts of GbnE relative to module 5.

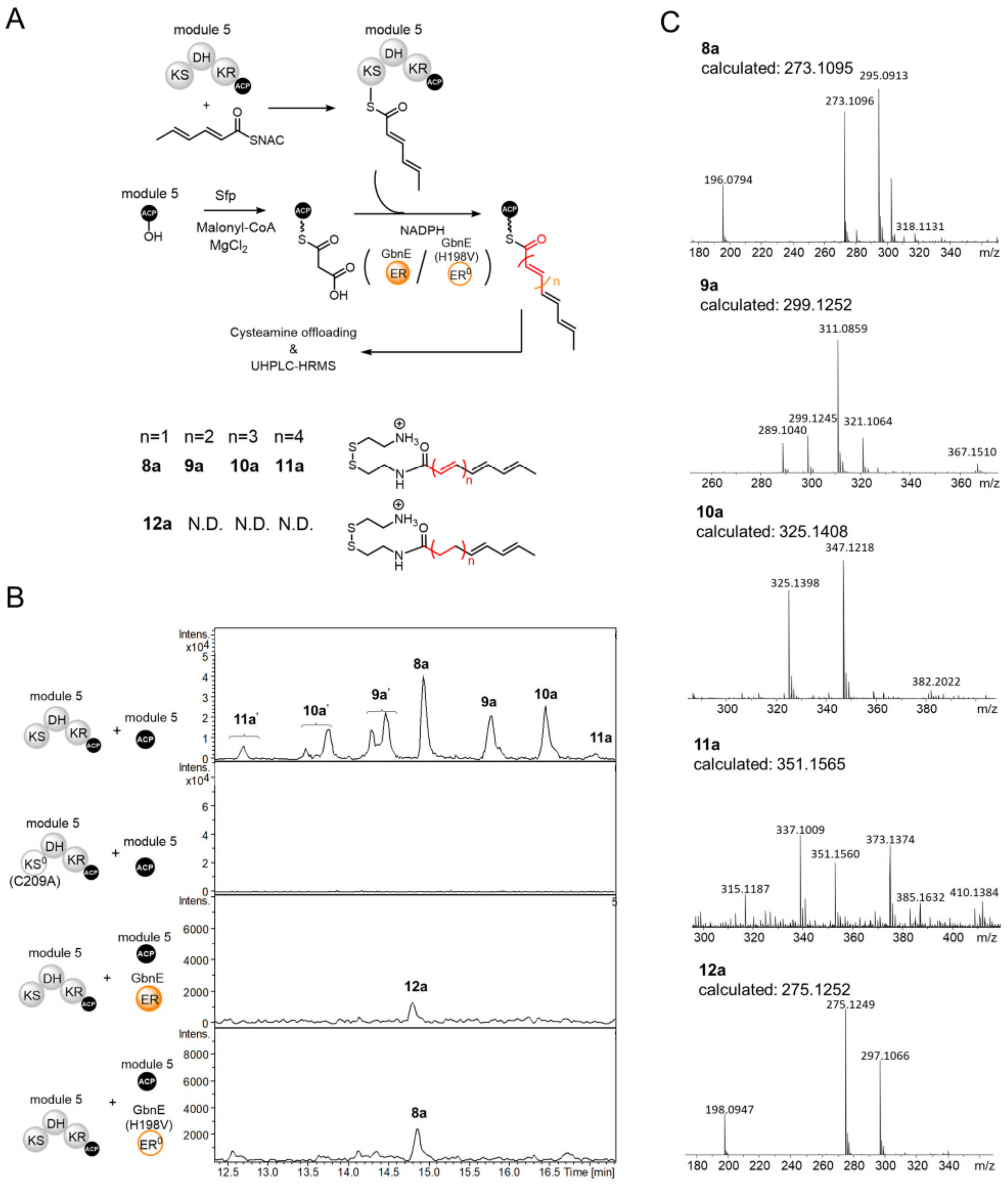

Figure S11. UHPLC-ESI-Q-TOF-MS analysis of the cysteamine offloading assays following module 5, module 5 + ACP5 domain and GbnE or GbnE(H198V) *in vitro* reconstitution. (A) Scheme illustrating the assay design. (B) Extracted ion chromatograms corresponding to *m/z* values of the $[M+H]^+$ ions for cysteamine offloading adducts **8a**, **9a**, **10a**, **11a** and **12a** from UHPLC-ESI-Q-TOF-MS analyses of reactions in the presence and absence of GbnE, and the presence of the H198V mutant of GbnE. No products are observed in control reaction lacking GbnE and employing a C209A mutant of module 5, which has a catalytically inactive KS domain. 9a', 10a' and 11a' are hypothesized to be the stereoisomers of **8a, 9a, 10a** and **11a** resulting from *E* to *Z* configurational isomerization of one or more double bond in the polyene. (C) Calculated and observed *m/z* values of **8a**, **9a**, **10a**, **11a** and **12a** are shown.

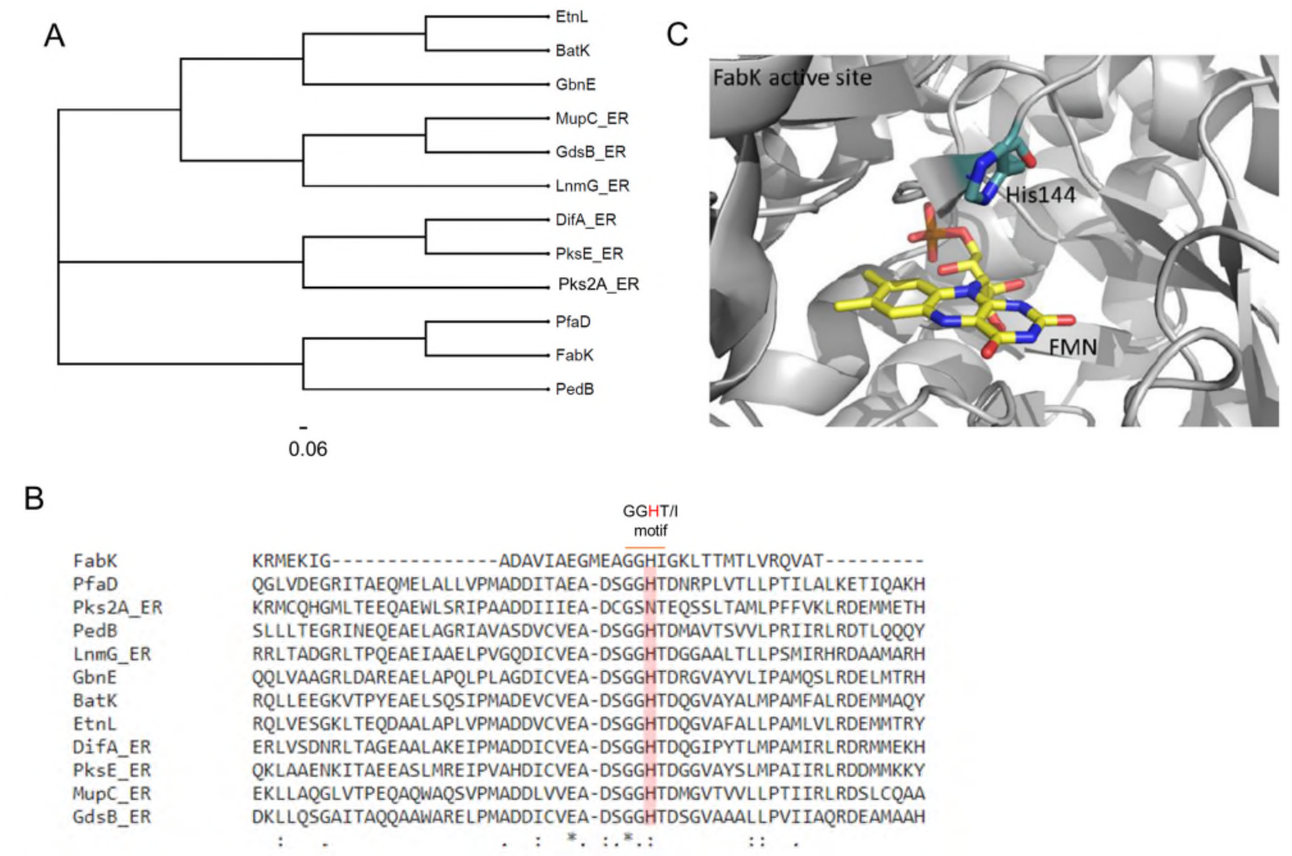

Figure S12. Phylogenetic analysis (A) and sequence alignment (B) of *trans*-acting ERs highlighting the conserved GGHT/I motif. The proposed catalytic residue (His144) for FabK, enoyl-ACP reductase from *Streptococcus pneumoniae,* is highlighted in red. (C) The structure of FabK from *Streptococcus pneumoniae* (PDB: 2Z6I) shows His144 is located in the active site close to the FMN cofactor. PfaD: ER from polyunsaturated fatty acid synthase in *Shewanella oneidensis* MR-1[9], Pks2A_ER: macrolactin[10], PedB: onnamide[11], LnmG_ER: leinamycin[12], GbnE: gladiolin[13], BatK: batumin/kalimatacin[14], EtnL: etnangien[15], DifA_ER: difficidin[16], PksE_ER: bacillaene/dihydrobacillaene[17], MupC_ER: mupirocin[18], GdsB_ER: gladiostatin[3].

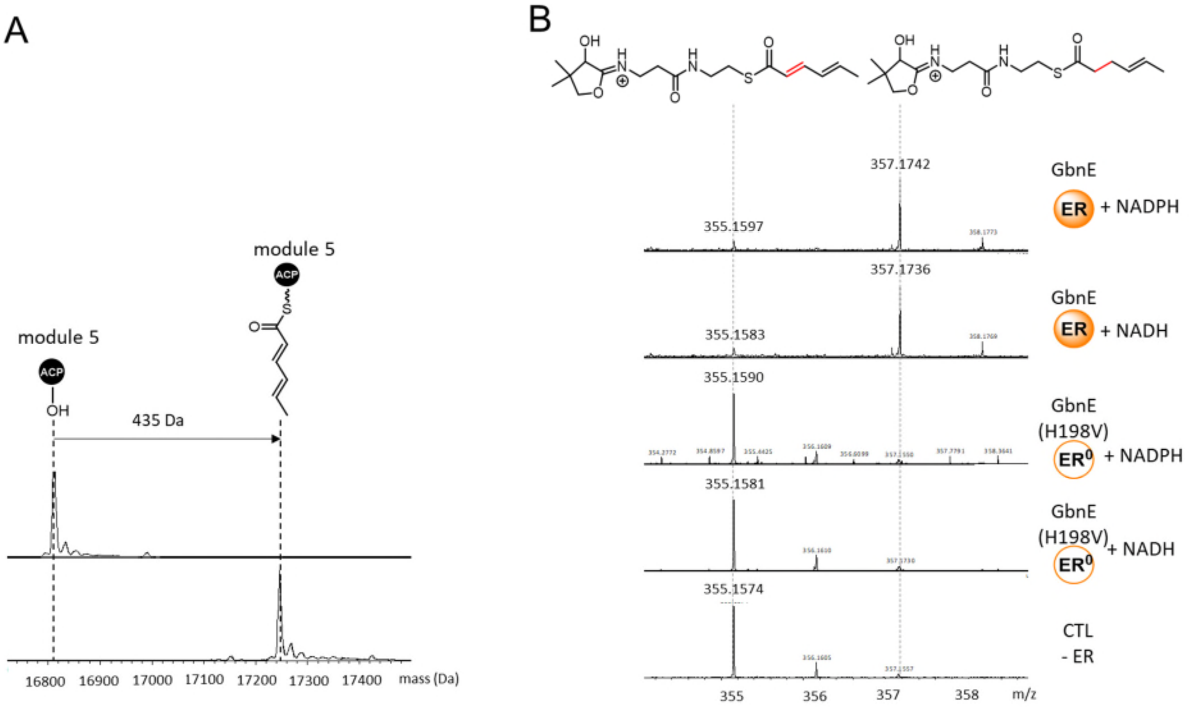

Figure S13. *In vitro* characterization and UHPLC-ESI-Q-TOF-MS of GbnE(H198V). (A) Deconvoluted mass spectra of the module 5 *apo*-ACP domain from the gladiolin PKS (top) following incubation with 2,4-hexadienoyl-pantetheine **17**, CoaA, CoaD, CoaE, sfp, $MgCl_2$ and ATP (bottom) confirming successful loading of the 2,4-hexadienoyl-Ppant unit. (B) Mass spectra of Ppant ejection ions from the 2,4-hexadienoylated-*holo*-ACP domain following incubation with GbnE and NADPH (top), GbnE and NADH (2nd), GbnE-H198V and NADPH (3rd), GbnE-H198V and NADH (4th) and no ER (bottom) confirming the enoyl reductase activity of GbnE-H198V is abolished. CTL: control.

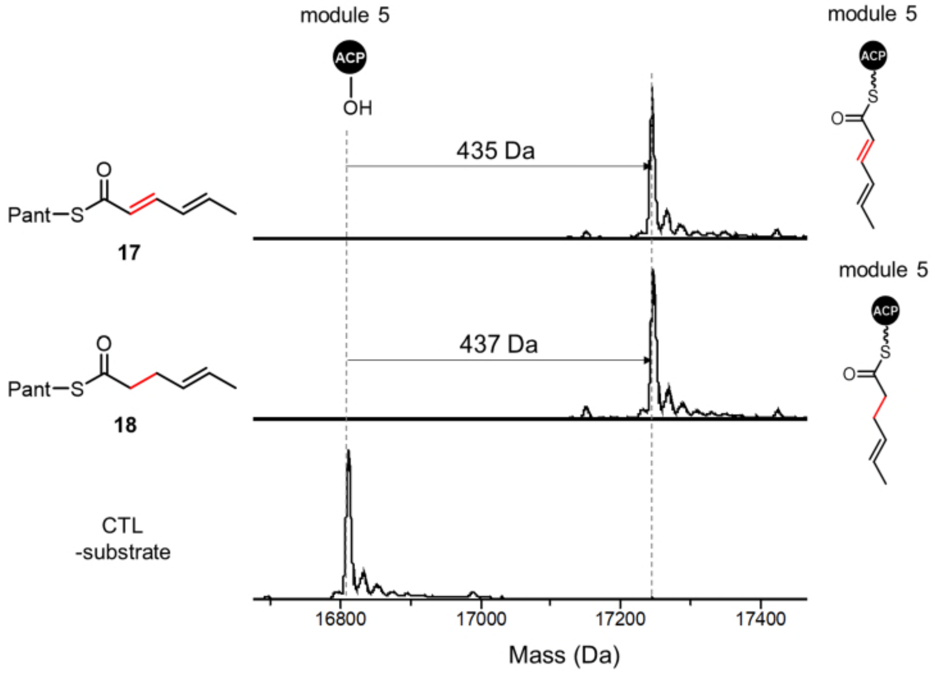

Figure S14. UHPLC-ESI-Q-TOF-MS analysis of 2,4-hexadienoyl-Ppant and 4-hexaenoyl-Ppant loading of module 5 ACP domain from gladiolin PKS. Deconvoluted mass spectra of module 5 ACP domain following incubation with 2,4-hexadienoyl-pantetheine **17** (top) and 4-hexaenoyl-pantetheine **18** (middle) in the presence of CoaC, CoaD, Sfp, $MgCl_2$ and ATP**,** CTL: control reaction, without substrate (bottom).

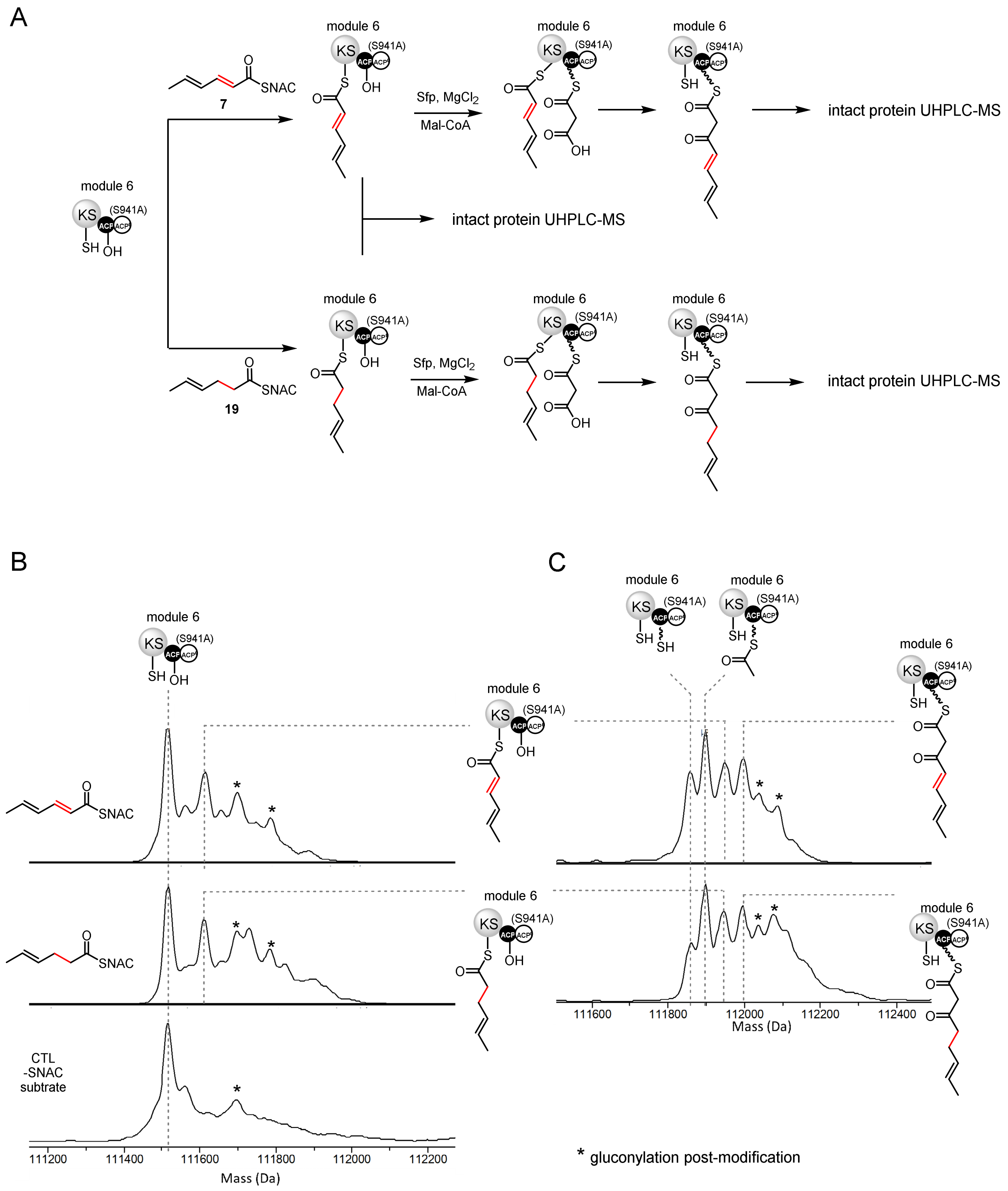

Figure S15. *In vitro* characterization and UHPLC-ESI-Q-TOF-MS analysis of substrate specificity of module 6 KS domain from the gladiolin PKS in the chain elongation reaction. (A) scheme illustrating module 6 *in vitro* chain elongation assay. (B) Deconvoluted mass spectra of module 6 (S941A) following incubation with 2,4-hexadienoyl-SNAC **7** (top) or 4-hexaenoyl-SNAC **19** (middle) or no substrate as control (bottom), CTL: control reaction. (C) Deconvoluted mass spectra of module 6 (KS-ACP-ACP-S941A), acylated with a 2,4-hexadienoyl (top) or 4-hexaenoyl (bottom) unit, following addition of Sfp, $MgCl_2$ and Malonyl-CoA.

Codon optimized DNA sequences

>*etnL*

ATGATTACCGCCAAAAGTCTGGGTGACCGCAGTTTCAAAGCCGACTATGGCGTGGATTACGCCTATGTGGCGGGTGCGATGTATAAA
GGCATCGCGAGCAAGGAGCTGGTTGTTGCCATGGGCAAAGCCGGTTTTATCGCCTATCTGGGTACCGGTGGTCTGGATGAACGCGA
AATCGAGGCGAGCATTCGCAGCATTCAGAGCGTTATCGCGGGCCGTGCGTACGGCATGAATCTGCTGAGCAATCTGGAAAGCCCGG
AACTGGAGGAGCGTACCATCGATCTGTATCTGCGTCACGGTGTTCGTTGTGTTGAGGCCGCCGCGTATATGCGCGTTACGCCAGCG
CTGGTGCGTTATCGCCTCCAAGGTCTGGCCAGTGGCGCGGGTCGCACGCTGGTTGCCCCACGTCGTGTTCTGGCGAAAGTTAGTC
GCCCGGAAGTTGCGGCCGCGTTTATGCAGCCAGCCCCGGAAGCGGTGGTTCGTCAGCTGGTTGAGAGTGGCAAACTGACCGAAC
AAGATGCCGCGCTGGCCCCACTGGTGCCAATGGCCGATGATGTGTGCGTGGAAGCCGACAGTGGTGGCCATACCGATCAAGGTGT
GGCCTTCGCGCTGCTGCCAGCCATGCTGGTTCTGCGCGACGAGATGATGACGCGCTACCGTTACGAGAAACGCATCCGCGTTGGT
GCGGCGGGTGGTATTGGTACCCCACACGCGGCCGCCGCCGCCTTTGTTATGGGTGCGGATTTCATTCTGACGGGCAGCATTAACCA
GTGCACCCGTGAGGCCGGTACGAGCGAACCAGTGAAAGCGCTGCTGCAGCATCTGAACGTTCAAGATACCACCTACGCCCCGGCC
GGTGACATGTTCGAACTGGGTAGCAAGATCCAAGTTGTTCGCCGTGGTCTGTTCTTTCCAGCCCGCGCCAACAAACTGCACGAACT
CTACATGCGCCACGGCAGCCTCGAAGAAATTGACGCGAAAACGCGCCAGCAGATTCAAGAAAAGTACTTTCGCCGCAGCTTCGATG
CGGTTTGGAGCGAAACCCGCAGCTACTATGCGCGTACGTACCCGCATCGTCTGGCCGAGATCGAACGCAGCCCGAAACAGAAAATG
GCGGCCGTGTTCCGTTGGTACTTTGCGCATACCACCCGTCTGGCGCTGGAAGGTGTTGAAGACCAGCGCCTCGACTACCAGATCCA
TTGTGGTCCAGCGCTGGGCGCGTTCAATCAGTGGACCAAGGGTACGGCCATTGAACCATGGCAGAATCGCTACGTGGCCGACATC
GCCCGCCGTATTATGGAGGGTACCGCGGATCTGCTGAACGCCCGCTTTGGCGCCATGAAGGAAGCGGCGGAACCGCCACAGAGC
GTTAGCAGTTAA

>region encoding Etn_module 5 ACP domain

AGTCCCGGTGATCTGGCGGCCGCCAGTCCAGCGAATGCCAGCACCCCAGCCATTCGCGAAGAAGCGCTGCGCCAAGTTAAAAAAT
TATTAGCGCCGGTGATCAAACTGCCGGTGGAACGCATCGATGCCGCCGCCAGCTTTGAAACCTACGGTATCGAAAGCGTGATGGCC
GTTGAACTGACCGATCGTTTAGAAGCGGTGTTCGGTCCGCTGAGCAAAACGCTGCTGTTCGAAGCGAAAACCGTGCGCGAACTGG
CCGATTACTTTGTGGAACATCATGCCGCCACTTTAGGCTCTTTACTGGGCGGTGCGACCGCCCCGGCCGCCCCGGCCACCGCCGT
GAGCGCC

Recombinant protein sequences

N-His$_6$-module 1 ER domain

```
MHHHHHHGKP IPNPLLGLDS TENLYFQGID PFT-
        10         20         30         40         50         60
AAPSPSGEAS TPLPAGDYGL VVRTVHALDE LSVEPWTPGE PGDDEVLIEV RASALNFPDV
        70         80         90        100        110        120
MCVQGLYPTQ PAYPFVPGFE VAGVVAAVGR AVVGIRVGEA VLALTGERMG GLASRVVVPA
       130        140        150        160        170        180
ANVLPKPSRL SFEEAASLPV AFLTAHHAFE TGRLAAGERV LIQTATGGCG LAAIQLARLR
       190        200        210        220        230        240
GARVYGTSSR AAKRALLERI GVEHVLDYRA AFDRELAGLT DGRGVDVVLN MLSGDAIQRG
       250        260        270        280        290        300
LDSLAPAGRY VEIAVHGLRT SGTLDLSRLV DNQSFHSIDL RRARARGLDW GETLAGLLPL
       310        320        330        340        350        360
FETGALVPIV SRVYPAEQLG EALRYVATGE HVGKVVIRHR GGALDDCVEA CVSRLVAQRE
       370        380        390
IAAREAARPA SLPVVPRRAS AAAQPASETH AP
```

N-His$_6$-module 1 ACP-ER didomain

```
MHHHHHHGKP IPNPLLGLDS TENLYFQGID PFT-

        10         20         30         40         50         60
PEPAEAGNVA VQATDADSFD LNISHDEIIE RSVAPDAATS RRRTIDSGPD LILERELAES
        70         80         90        100        110        120
LGQSLYLPAD EIDPERPFVE LGLDSIVGVE WARAINKAYG IALPATRLYD HVTIRLLAAH
       130        140        150        160        170        180
LVEQWGVARR DSARPAEPPV GLVAPVTRAP IAAAPSPSGE ASTPLPAGDY GLVVRTVHAL
       190        200        210        220        230        240
DELSVEPWTP GEPGDDEVLI EVRASALNFP DVMCVQGLYP TQPAYPFVPG FEVAGVVAAV
       250        260        270        280        290        300
GRAVVGIRVG EAVLALTGER MGGLASRVVV PAANVLPKPS RLSFEEAASL PVAFLTAHHA
       310        320        330        340        350        360
FETGRLAAGE RVLIQTATGG CGLAAIQLAR LRGARVYGTS SRAAKRALLE RIGVEHVLDY
       370        380        390        400        410        420
RAAFDRELAG LTDGRGVDVV LNMLSGDAIQ RGLDSLAPAG RYVEIAVHGL RTSGTLDLSR
       430        440        450        460        470        480
LVDNQSFHSI DLRRARARGL DWGETLAGLL PLFETGALVP IVSRVYPAEQ LGEALRYVAT
       490        500        510        520        530        540
GEHVGKVVIR HRGGALDDCV EACVSRLVAQ REIAAREAAR PASLPVVPRR ASAAAQPASE

THAP
```

N-His6-SUMO-GbnE

```
MGSSHHHHHH GSGLVPRGSA SMSDSEVNQE AKPEVKPEVK PETHINLKVS DGSSEIFFKI
KKTTPLRRLM EAFAKRQGKE MDSLRFLYDG IRIQADQTPE DLDMEDNDII EAHREQIGG-
        10         20         30         40         50         60
MAMITASSLG NADFRNDYRI KYAYLAGAMY RAIASKELVV ALGKAGLMGF LGTGGLRLDD
        70         80         90        100        110        120
IEAAILQIQA ELGVDGVYGM NLLADLERPE REEATVDLYL RHGVRHVEAA AYMRVTPALV
       130        140        150        160        170        180
RYRLRGASIG ADGRAVARHH VVAKVSRPEV ASQFMQPAPP ALVQQLVAAG RLDAREAELA
       190        200        210        220        230        240
PQLPLAGDIC VEADSGGHTD RGVAYVLIPA MQSLRDELMT RHRYAKRIRI GAAGGIGTPQ
       250        260        270        280        290        300
AAAAAFVMGA DFIVTGSINQ CTREAGTSDV VKALLQELDV QDTAYAPAGD MFELGAKVQV
       310        320        330        340        350        360
ARRGLFFAAR ASRLHELYQQ HASLDEIDPA MRSQIEQKFF RRGFDEIWDE TRRHYASSRP
       370        380        390        400        410        420
DQIAEIERSP KKKMAAVFRW YFAHSTQLAL SGDDTRLTDF QVHCGPALGA FNRWVRGTPL
       430        440        450        460
ESWQNRHVAD LAERIMQGTA AWLEARLASM ADSGGQRPSL QLQRQ
```

N-His6-module 3 ACP domain

```
MGSSHHHHHH SSGLVPRGSHM-

        10         20         30         40         50         60
TATVPTTVTT PTATENDALA ARAIEYFRKW LAAQLKVGAD QLDDDAPLDR YGIDSVRVMQ
        70         80         90        100        110        120
LTSALESRFG PLSKTLFFEY RNVAELSRHF VQAHRERMLD LLGLASPSAA PLPTSRPAGP
       130
APRPVLSSGP AI
```

N-His6-moudel 5 ACP domain

```
MHHHHHHGKP IPNPLLGLDS TENLYFQGID PFT-

        10         20         30         40         50         60
PNPASLSSVP GGQDATRAAS AALLADEALR HVKRQLAAVI RLPVERIDED ASFEEYGIDS
        70         80         90        100        110        120
VMAVELTDRL ERACGPLSKT LLFEYQSVRD LTAFLVRHHA EGLGAALGLG EASSDGETAK
AEV
```

N-His6-moduel 10 ACP domain

```
MGSSHHHHHH SSGLVPRGSHM-
```

```
        10         20         30         40         50         60
AQAKPVAGAA PAAAGVPAPA PIADAAELGA RVDAALARAV CEILRIGESD VDAETDFSAY
        70         80         90        100        110        120
GFDSISLTEF SNRIGERLGV ELLPTIFYEY PSLLALRGFL LAEHGAALAA ALQVQPAAGR
       130        140
QVEPDPEPHR ESNRESNPGA AAVGLPEG
```

N-His$_6$-moudel 13 ACP domain

```
MHHHHHHGKP IPNPLLGLDS TENLYFQGID PFT-
        10         20         30         40         50         60
VAAGYDGARA AALAAGESTR ASFDEALRRF VTDQLAAQGV ALAGRLGDDT PFFDAGLDST
        70         80         90        100        110
HLLALVRALE THCGRTFYPT LLFEHQTLRE LAAHLHRETP AAFGQAVPVW SESVAA
```

N-His$_6$-module 5

```
MGSSHHHHHH SSGLVPRGSHMAS-
        10         20         30         40         50         60
AGARTMRAMA VATRGLAVPP ATASASLPQD RPEATATGTQ AVAVIGLAGR YPQAADLDAF
        70         80         90        100        110        120
WENLSTGRDC ITEIPSTRWD HEVYFDARKG QPGKSYSKWG GFLDGVDEFD PSFFSISPRE
       130        140        150        160        170        180
AQLMDPQERL FLQCAYHALE DAGHTRASLG AARVGVFVGV MYEEYPYHGS PSQGTTQPQA
       190        200        210        220        230        240
LGGSSASIAN RVSYAFNLNG PSIAMDTMCS SSLTALHLAC QSLRLGECEL ALAGGVNVSI
       250        260        270        280        290        300
HPNKYLGLSQ GQFASSEGRC RSFGAGGDGY VPSEGVGCVL LRPLAAAEAA GDRILGVIRA
       310        320        330        340        350        360
SAINHGGRTN GYTVPNPNAQ GELIAEALRA SGVDARAISY LEAHGTGTAL GDPIEIAGLV
       370        380        390        400        410        420
KAYGAWEGEP GEPGEPGDAR LEPCAIGSVK SNIGHCESAA GIAGLTKVLL QMRHGKLAPS
       430        440        450        460        470        480
LHAQTLNPLI DFGRTPFRVQ RELAPWRRPR VRVDGVEREM PRLAGISSFG AGGANAHVIV

       490        500        510        520        530        540
EEYVARAVEA ADARREGQPA IVVLSARSEA QVLIQARNLQ AAIEREAYGE GELAALAHTL
       550        560        570        580        590        600
QAGREAFEVR LATTVTSMAM LVERLASLAG EAPDYSAWMR GETRRDAADL PAPETVEGWL
       610        620        630        640        650        660
AQGRVEPLLR AWLDGLAFDW RRLRAADPPI RPLRLPGYPF ARQRYWADPP AAAAPRVARL
       670        680        690        700        710        720
HPLLHENRST AFEPQFVSHF DGREACLADH RVAHARVLPG AAQLEIARAA AALSLGEAAA
```

```
       730        740        750        760        770        780
LELTEVAWLQ PACFDEQGGS LRIALFVDSE TEAEFDIGSL DGDTVYSQGR LRLREAASPV
       790        800        810        820        830        840
VLELASLRAA CGQVPLAPEA LYASFAAAGL QYGPAHRAIA ELRTGLDTAG RPQVLARLEV
       850        860        870        880        890        900
PDGAADEALV LHPSLVDGAI QATIGLLLVP GGARRAMLPA RLGRVGVAAA TPARGWAWLR
       910        920        930        940        950        960
FAEGSGPEDV APRIDLSICD EHGAVALEIE ALTLMPAPVA SQTGVETLWL APAWAEAEAP
       970        980        990       1000       1010       1020
LDAPRGAAQP TREVFVAGQL PEGVLAALAR RYGESRVHVA PGADAMPLAA GYVAAADALF
      1030       1040       1050       1060       1070       1080
AIVRERLADV ASGECLFQLL RVEDGTAGAA CLDGLGALLR TASIENPRFA TQSIRIDAAD
      1090       1100       1110       1120       1130       1140
ATDGETLLAL LDANARERDS REIAYLDGRR RQRRHLELSQ PGGDVPAWPA GTVALITGGL
      1150       1160       1170       1180       1190       1200
GGIGYRVAES IAASGPGTTL LLCGRSEPAD AAARLGALRA AGANAEFVRT DITDAAATEA
      1210       1220       1230       1240       1250       1260
LVAGIVARHG RLDTVIHSAG IVRDNFVIRK NASELHAVLA PKVAGLVNLD AATRSLPLRE
      1270       1280       1290       1300       1310       1320
LIVFSSVSGA FGNAGQADYA CANAFMDAFA AWRATRVAAG ERSGRTLSIG WPLWAEGGMR
      1330       1340       1350       1360       1370       1380
VDAAVEAKLR RDGLAPLDTA SGLAVLARCR QLPPELTQVV VLAGDAARLR ARHGLAAAAI
      1390       1400       1410       1420       1430       1440
GAAASAPNRA PNPAPNPASL SSVPGGQDAT RAASAALLAD EALRHVKRQL AAVIRLPVER
      1450       1460       1470       1480       1490       1500
IDEDASFEEY GIDSVMAVEL TDRLERACGP LSKTLLFEYQ SVRDLTAFLV RHHAEGLGAA
      1510
LGLGEASSDG ETAKAEV
```

N-His6-module 6

```
MGSSHHHHHH SSGLVPRGSHMAS-
        10         20         30         40         50         60
DGETAKAEVA VAALAAGAVA TARAAGPALA PVAPRTRRRP RGRLPGRESE PPRITAIAVI
        70         80         90        100        110        120
GLAGRYPQAA DLDAFWENLS TGRDCITEIP STRWDHEAYF DARKGQPGKS YSKWGGFLDG
       130        140        150        160        170        180
VDEFDPFFFN ISPREAQLMD PQERLFLQCA YHALEDAGHT RASLGAVRVG VFVGVMYEEY
       190        200        210        220        230        240
HLLSDPAGGD LAQTYIPGGY LSSVANRVSY FGNFRGPSFG VDTMCSSSLT ALHLACQSLR
       250        260        270        280        290        300
LGECELALAG GVNVSIHPNK YLGLSQGQFA SSEGRCRSFG AGGDGYVPSE GVGCVLLRPL
       310        320        330        340        350        360
AAAEAAGDRI LGVIRASAIN HGGRTNGYTV PNPNAQGELI AEALRASGVD ARAISYLEAH
```

```
       370        380        390        400        410        420
GTGTALGDPI EIAGLVKAYG AWEGEPGEPG DARLEPCAIG SVKSNIGHCE SAAGIAGLTK
       430        440        450        460        470        480
VLLQMRHGKL APSLHAQTLN PLIDFGRTPF RVQRELAPWR RPRVRVDGVE REMPRLAGIS
       490        500        510        520        530        540
SFGAGGANAH LIVEEYVARA VEAADARREG QPAIVVLSAR SEAQVLIQAR NLQAAIAREA
       550        560        570        580        590        600
YGEGELAALA HTLQAGREAF EVRLATTVTS MAMLVERLAS LAGETPDYSA WMRGEARRDG
       610        620        630        640        650        660
NDALAHFAKD PELNEVLRKW LRAGNPVRIA ELWTRGLDID WAAMQEDGPA PARLRLPGYP
       670        680        690        700        710        720
FATKRYWMAR ADGSPALAAR AAVPARPEPP TRAAVPPAAV KVLAEVLADA PRVAIVLGEP
       730        740        750        760        770        780
GGFEARTEGA ARAASIRLAV LDPLDEFEPP AAPAAARQDD GQTAAAARQV PAGIEVSIER
       790        800        810        820        830        840
LRLSLAQMLC AEAADLGADV PFGELGLDSV VGVEWTRAIG REYGVTLAAA TLYDHPTLTT
       850        860        870        880        890        900
LAAWLAATVE AGAAIATAGR DVPPRPDAHP VRLVEPDQDV ATPLAAAAAV EGGNEAEVAA
       910        920        930        940        950        960
GMAMPIEALV ERLAGTLAQA LCAEREEIDP DTPFAELGID SVVGVEWVRD INRAFGTELK
       970        980        990       1000       1010       1020
ATVLFDHATV KLLAAHLAPL ACPAPHSVRV DPPAAPVEQL AAASAVPARE LADTARHAGS
      1030       1040
RGHVQAVEPA APVAKTGNAP
```

N-His_6-MBP-EtnL

```
MGSSHHHHHHSSGLVPRGSHMASGKIEEGKLVIWINGDKGYNGLAEVGKKFEKDTGIKVTVEHPDKLEEKFPQVAATGDG

PDIIFWAHDRFGGYAQSGLLAEITPDKAFQDKLYPFTWDAVRYNGKLIAYPIAVEALSLIYNKDLLPNPPKTWEEIPALD

KELKAKGKSALMFNLQEPYFTWPLIAADGGYAFKYENGKYDIKDVGVDNAGAKAGLTFLVDLIKNKHMNADTDYSIAEAA

FNKGETAMTINGPWAWSNIDTSKVNYGVTVLPTFKGQPSKPFVGVLSAGINAASPNKELAKEFLENYLLTDEGLEAVNKD

KPLGAVALKSYEEELAKDPRIAATMENAQKGEIMPNIPQMSAFWYAVRTAVINAASGRQTVDEALKDAQTRITKGENLYF

QGS-
        10         20         30         40         50         60
MITAKSLGDR SFKADYGVDY AYVAGAMYKG IASKELVVAM GKAGFIAYLG TGGLDEREIE
        70         80         90        100        110        120
ASIRSIQSVI AGRAYGMNLL SNLESPELEE RTIDLYLRHG VRCVEAAAYM RVTPALVRYR
       130        140        150        160        170        180
LQGLASGAGR TLVAPRRVLA KVSRPEVAAA FMQPAPEAVV RQLVESGKLT EQDAALAPLV
       190        200        210        220        230        240
PMADDVCVEA DSGGHTDQGV AFALLPAMLV LRDEMMTRYR YEKRIRVGAA GGIGTPHAAA
```

250 260 270 280 290 300

AAFVMGADFI LTGSINQCTR EAGTSEPVKA LLQHLNVQDT TYAPAGDMFE LGSKIQVVRR

310 320 330 340 350 360

GLFFPARANK LHELYMRHGS LEEIDAKTRQ QIQEKYFRRS FDAVWSETRS YYARTYPHRL

370 380 390 400 410 420

AEIERSPKQK MAAVFRWYFA HTTRLALEGV EDQRLDYQIH CGPALGAFNQ WTKGTAIEPW

430 440 450

QNRYVADIAR RIMEGTADLL NARFGAMKEA AEPPQSVSS

N-His$_6$-Etn module 5 ACP domain

MGSSHHHHHHSSGLVPRGSHM-

10 20 30 40 50 60

SPGDLAAASP ANASTPAIRE EALRQVKKLL APVIKLPVER IDAAASFETY GIESVMAVEL

70 80 90 100 110

TDRLEAVFGP LSKTLLFEAK TVRELADYFV EHHAATLGSL LGGATAPAAP ATAVSA

N-His$_6$-Etn module 10_ACP domain

MGSSHHHHHHSSGLVPRGSH-

10 20 30 40 50 60

TPASEQPAPL ADGAGEASGL RDLVERTLVR AVSRVLKIHE ADIELDAELS AFGFDSLSLT

70 80 90 100 110

ELSNRLSAEL GVELLPTVFF EHPSLAALET FLLATHLGAL ARTFRAEVAA

N-His$_8$-EtnK_AT

MKHHHHHHHH GGLVPRGSHGSH-

10 20 30 40 50 60

AGPRATVAFM FPGQGSQKRG MGAGLFDSVP EYAAVEREVD ALLGYSMRAL CHEDPDGRLK

70 80 90 100 110 120

ETQYTQPALY VVNALHYYDA IARGQRPDYV AGHSLGEYNA LLAAGAFDLL TGLRLVKKRG

130 140 150 160 170 180

ELMAASVSGG MAAVIGMDEG RIKQVLTENG LGTIDVANFN SPSQIVISGP VADIARGHTV

190 200 210 220 230 240

FEDAGARTYV ILPVSAAFHS RYVEETGRAF ADFIAPMRFE ALRIPVISNV SARPYEAKDP

250 260 270 280 290

SATIKSLLVQ QITRPVQWVQ SVSFLMAQGV KEFREIGPGN VLTRLVQQIE RQPSGA

References

(1) Luis E. N. Quadri, Paul H. Weinreb, Ming Lei, Michiko M. Nakano, Peter Zuber, and Christopher T. Walsh. Characterization of Sfp, a *Bacillus subtilis* phosphopantetheinyl pransferase for peptidyl parrier protein pomains in peptide pynthetases. *Biochemistry* **1998**, 37 (6), 1585–1595.

(2) Agarwal, V.; Diethelm, S.; Ray, L.; Garg, N.; Awakawa, T.; Dorrestein, P. C.; Moore, B. S. Chemoenzymatic synthesis of acyl coenzyme a substrates enables *in situ* labeling of small molecules and proteins. *Org. Lett.* **2015**, *17* (18), 4452–4455.

(3) Flannagan, R. S.; Linn, T.; Valvano, M. A. A system for the construction of targeted unmarked gene deletions in the genus *Burkholderia*. *Environ. Microbiol.* **2008**, *10* (6), 1652–1660.

(4) Nakou, I. T.; Jenner, M.; Dashti, Y.; Romero-Canelón, I.; Masschelein, J.; Mahenthiralingam, E.; Challis, G. L. Genomics-driven discovery of a novel glutarimide antibiotic from *Burkholderia gladioli* reveals an unusual polyketide synthase chain release mechanism. *Angew. Chem. Int. Ed.* **2020**, *59* (51), 23145–23153.

(5) Hobson, C.; Jenner, M.; Jian, X.; Griffiths, D.; Roberts, D. M.; Rey-Carrizo, M.; Challis, G. L. Diene Incorporation by a dehydratase domain variant in modular polyketide synthases. *Nat. Chem. Biol. 2022 1812* **2022**, *18* (12), 1410–1416.

(6) O'Duill, M. L.; Matsuura, R.; Wang, Y.; Turnbull, J. L.; Gurak, J. A.; Gao, D.-W.; Lu, G.; Liu, P.; Engle, K. M. Tridentate directing groups stabilize 6-membered palladacycles in catalytic alkene hydrofunctionalization. *J. Am. Chem. Soc.* **2017**, *139* (44), 15576–15579.

(7) Geoghegan K. F.; Dixon H. B. F.; Rosner P.J.; Hoth L. R.; Lanzetti A. J.; Borzilleri K. A.; Marr E. S.; Pezzullo L. H.; Martin L. B.; LeMotte P K.; McColl A. S.; Kamath A. V; and Stroh J. G. Spontaneous α-*N*-6-Phosphogluconoylation of a "His Tag" in *Escherichia coli*: The cause of extra mass of 258 or 178 Da in fusion proteins. *Anal. Biochem.* **1999**, *267* (1), 169-184.

(8) Giglione, C.; Boularot, A.; Meinnel, T. Protein N-terminal methionine excision. *Cellular and Molecular Life Sciences*. **2004**, *61* (12), 1455–1474.

(9) Lee, S. J.; Jeong, Y. S.; Kim, D. U.; Seo, J. W.; Hur, B. K. Eicosapentaenoic Acid (EPA) Biosynthetic Gene Cluster of Shewanella Oneidensis MR-1: Cloning, Heterologous expression, and effects of temperature and glucose on the production of EPA in *Escherichia Coli*. *Biotechnol. Bioprocess Eng.* **2006**, *11* (6), 510–515.

(10) Schneider, K.; Chen, X. H.; Vater, J.; Franke, P.; Nicholson, G.; Borriss, R.; Süssmuth, R. D. Macrolactin Is the Polyketide biosynthesis product of the Pks2 cluster of *Bacillus amyloliquefaciens* FZB42. *J. Nat. Prod.* **2007**, *70* (9), 1417–1423.

(11) Piel, J.; Hui, D.; Wen, G.; Butzke, D.; Platzer, M.; Fusetani, N.; Matsunaga, S. Antitumor polyketide biosynthesis by an uncultivated bacterial symbiont of the marine sponge *Theonella swinhoei*. *Proc. Natl. Acad. Sci. U. S. A.* **2004**, *101* (46), 16222–16227.

(12) Tang, G. L.; Cheng, Y. Q.; Shen, B. Leinamycin biosynthesis revealing unprecedented architectural complexity for a hybrid polyketide synthase and nonribosomal peptide synthetase. *Chem. Biol.* **2004**, *11* (1), 33–45.

(13) Song, L.; Jenner, M.; Masschelein, J.; Jones, C.; Bull, M. J.; Harris, S. R.; Hartkoorn, R. C.; Vocat, A.; Romero-Canelon, I.; Coupland, P.; Webster, G.; Dunn, M.; Weiser, R.; Paisey, C.; Cole, S. T.; Parkhill, J.; Mahenthiralingam, E.; Challis, G. L. Discovery and biosynthesis of gladiolin: A *Burkholderia gladioli* antibiotic with promising activity against *Mycobacterium tuberculosis*. *J. Am. Chem. Soc.* **2017**, *139* (23), 7974–7981.

(14) Uytterhoeven, B.; Lathouwers, T.; Voet, M.; Michiels, C. W.; Lavigne, R. A Protein interaction map of the kalimantacin biosynthesis assembly line. *Front. Microbiol.* **2016**, *7* (NOV), 1726.

(15) Menche, D.; Arikan, F.; Perlova, O.; Horstmann, N.; Ahlbrecht, W.; Wenzel, S. C.; Jansen, R.; Irschik, H.; Müller, R. Stereochemical determination and complex biosynthetic assembly of etnangien, a highly potent rna polymerase inhibitor from the *Myxobacterium Sorangium cellulosum*. *J. Am. Chem. Soc.* **2008**, *130* (43), 14234–14243.

(16) Wagner, D. T.; Zeng, J.; Bailey, C. B.; Gay, D. C.; Yuan, F.; Manion, H. R.; Keatinge-Clay, A. T. Structural and Functional Trends in Dehydrating Bimodules from Trans-Acyltransferase Polyketide Synthases. *Structure* **2017**, *25* (7), 1045-1055.

(17) Bumpus, S. B.; Magarvey, N. A.; Kelleher, N. L.; Walsh, C. T.; Calderone, C. T. Polyunsaturated fatty-acid-like *trans*-enoyl reductases utilized in polyketide biosynthesis. *J. Am. Chem. Soc.* **2008**, *130* (35), 11614–11616.

(18) El-Sayed, A. K.; Hothersall, J.; Cooper, S. M.; Stephens, E.; Simpson, T. J.; Thomas, C. M. Characterization of the mupirocin biosynthesis gene cluster from *Pseudomonas fluorescens* NCIMB 10586. *Chem. Biol.* **2003**, *10* (5), 419–430.
